# Human leukocyte extract enhances albendazole efficacy against *Echinococcus multilocularis* in mice by reducing parasitic microRNAs, myeloid and lymphoid immunosuppressive cells

**DOI:** 10.1371/journal.pntd.0014591

**Published:** 2026-08-14

**Authors:** Gabriela Hrčková, Emília Dvorožňáková, Dagmar Mudroňová, Zuzana Jurčacková, Terézia Mačak Kubašková, Miroslava Petrová, Annamaria Bardelčíková

**Affiliations:** 1 Institute of Parasitology, Slovak Academy of Sciences, Košice, Slovakia; 2 Department of Microbiology and Immunology, University of Veterinary Medicine and Pharmacy, Košice, Slovakia; 3 Department of Pharmacology, Faculty of Medicine, Pavol Jozef Šafarik University, Košice, Slovakia; Xuzhou Medical University, CHINA

## Abstract

**Background:**

*Echinococcus multilocularis* induces a serious zoonotic infection in humans and growth of metacestodes is associated with immunosuppression and tolerance. Current treatment relies almost exclusively on albendazole, a parasitostatic drug with variable clinical efficacy. Human low molecular weight leukocyte extract (HLE) has been shown to modulate innate and adaptive immunity at various diseases and can serve as promising and safe adjuvant to chemotherapy.

**Methodology:**

In the present study we investigated the effects of albendazole (ABZ), human leukocyte extract (HLE) and their combination on murine alveolar echinococcosis, developing in the peritoneal cavities, by monitoring cysts weight, levels of circulating parasitic miRNAs, cytokine levels by ELISA and qPCR, the proportions of lymphoid and myeloid cells in spleens and peritoneal cavities by flow cytometry and proliferation of T and B lymphocytes and NO production ex vivo.

**Principal findings:**

Albendazole therapy moderately reduced cyst mass, selectively altered circulating parasite-derived miRNA levels in line with moderate decrease of IL-10 and TGF-β and myeloid-derived suppressor cells without the effects on suppressed T and B cells proliferation and high NO production. HLE in combination with ABZ significantly reduced peritoneal cyst growth and these two therapies markedly decreased circulating parasite derived microRNAs and expression of T- and B-regulatory cells lineage-specific transcription factors. These molecular changes were accompanied by a marked restoration of T and B lymphocyte proliferation, significant decline of regulatory IL-10 and TGF-β cytokines and immunosuppressive myeloid cells and MHC II^low^ (M2) macrophages in spleens and peritoneal cavities, where HLE and ABZ + HLE interventions suppressed NO production and altered cytokine expression. The effect probably involved NF-κB downregulation, preventing excessive inflammation while enhancing ABZ efficacy.

**Conclusions:**

HLE as adjuvant enhanced albendazole efficacy against *Echinococcus multilocularis* infection in mice via reduction of T regulatoty type of response and MDSC expansion and shift of systemic and local immunity toward balanced Th1/Th2- biased profiles. The marked reduction in circulating parasite-derived miRNAs identifies a previously unrecognised mechanism that may contribute to the immunomodulatory effects of HLE and highlights these molecules as potential biomarkers of therapeutic response. Collectively, these results propose HLE as a promising adjunct immunotherapeutic candidate capable of counteracting parasite-induced immunosuppression and potentiating benzimidazole chemotherapy.

## Author summary

Alveolar echinococcosis (AE) is a life-threatening parasitic disease caused by the cestode *Echinococcus multilocularis*. Current treatment relies almost exclusively on albendazole, which inhibits parasite growth although therapeutical efficacy varies considerably among patients. The parasite profoundly suppresses the host immune response through the secretion of immunomodulatory molecules, including microRNAs, which may contribute to poor treatment outcomes. In this study, we investigated the mechanisms by which a human low-molecular-weight leukocyte extract (HLE), prepared from white blood cells of healthy donor´s by ultrafiltration, enhances host immune response and improves the efficacy of albendazole. Using a murine model of severe peritoneal AE at the subchronic stage of infection, we demonstrated that HLE markedly enhanced the antiparasitic efficacy of albendazole, resulting in a marked reduction in parasite burden. Circulating levels of four parasite-derived miRNAs were substantially decreased following HLE monotherapy and combination treatment, whereas no comparable reduction was obsertved with albendazole alone. These finding suggest that HLE may interfere with parasite-derived regulatory mechanisms involved in immune evasion. Furthermore, HLE profoundly reshaped both systemic and local immune responses by promoting protective Th1- and Th2-associated cytokine production, suppressing regulatory T-cell pathways exploited by the parasite to induce immune tolerance, and restoring T- and B- lymphocyte activity. HLE also markedly reduced the expansion of myeloid- derived suppressor cells which are known to contribute to parasite persistance through their immunosuppressive functions. Collectively, these findings demonstrate that immunomodulation with HLE can alleviate parasite-induced immunosuppression and thereby significantly nhance the antiparasitic activity of albendazole. This combination strategy represents a promising therapeutic approach for the therapy of this neglected and often fatal parasitic disease.

## Introduction

Alveolar echinococcosis (AE), although relatively rare, is one of the most severe parasitic zoonoses recorded in 47 endemic countries across the Northern Hemisphere and imposing a substantial socioeconomic consequences [1,2]. It has been estimated that approximatelly 10,489 new AE cases occur annually worldwide, with nearly 92% reported in China. In Slovakia, 137 cases of AE have been confirmed so far, with a mean age at diagnosis of 52 years [3]. Humans can be accidentally infected through ingestion of *E. multilocularis* eggs shed by foxes and other carnivores serving as definitive hosts [1]. In humans, AE is characterised by chronic and progressive hepatic damage resulting from infiltrative, tumour-like growth of metacestodes, which may occasionally disseminate to other organs. The early stages of infection are typically asymptomatic or associated with non-specific clinical manifestations, frequently resultings in delayed diagnosis and subsequent disease-related complications.

In experimental murine models, intraperitoneal inoculation with larval stages (protoscoleces) leads to the development of cysts primarily within the peritoneal cavity with possible later infiltration into the liver in advanced stages of infection. The curent treatment relies on radical surgical intervention combined with benzimidazole chemotherapy. Albendazole and mebendazole remain the only drugs currently recommended for treatment of AE. Although generally well tolerated, both drugs exhibit poor aqueous solubility, limited oral bioavailability and an absorption rate of albendazole is approximately 0.4% following a 400 mg oral dose 0–15 mg/kg body weight [4]. Consequently, these drugs exert predominantly parasitostatic rather than parasitocidal activity. In patients with inoperable disease, benzimidazole chemotherapy is usually administered lifelong, increasing the risk of adverse effects, including nausea, alopecia, leukopenia and further aggravation of immunosuppression [5,6].

Host immunity plays pivotal role in determining the clinical outcome of AE, yet the interplay between host immune responses and benzimidazole treatment remains incompletely understood [7]. Several strategies aimed at improving therapeutic efficacy are currently being investigated in experimental models, with immunotherapy emerging as a particularly promising adjunct to chemotherapy. For example, combined administration of the Th1-associated cytokines IL-12 and IFN-γ with albendazole was more effective in treating cystic echinococcosis in mice compared with albendazole monotherapy [8]. Similarly, Wang et al. [9] demonstrated that blockade of the *Echinococcus multilocularis* PD-1/PD-L1 signalling pathway, a key mechanism of parasite-induced immunosuppression, enhanced protective host immune responses and promoted immune-mediated control of disease progression. In combination with albendazole, this strategy may therefore represent an promising therapeutic approach. Likewise, co-administration of liposome-encapsulated muramyl tripeptide or glucan with albendazole resulted in a stronger and sustained inhibition of *E. multilocularis* cyst growth within the peritoneal cavity of mice than albendazole alone [10,11]. A synergistic inhibitory effect has also been reported in cystic echinococcosis following combined treatment with albendazole and the antidiabetic drug metformin, resulting in greater therapeutic efficacy than either treatment alone [12].

Our recent studies using a murine model of infection with the larval stages of the closely related cestode *Mesocestoides vogae* demonstrated that treatment with albendazole combined with the immunomodulator human low-molecular-weight leukocyte extract (HLE) significantly attenuated T- regulatory cells (Treg)- mediated immunosuppression while promoting a balanced Th1/Th2 innate and adaptive immune response, accompanied by more favourable cytokine and antibody profiles [13–15]. In addition to enhancing the therapeutic efficacy of albendazole, HLE co-administration partially reversed the persistence of immunosuppression observed in the spleen and peritoneal cavity following albendazole monotherapy.

Low-molecular-weight leukocyte extracts represent heterogeneous mixtures of substances with molecular weights bellow 10–12 kDa, isolated from mononuclear white blood cells or tissue leukocytes from non-immunised human or animal donors. Initially prepared by dialysis, and more recently by ultrafiltration, these extracts contain a broad spectrum of biologically active compounds, including cyclic nucleotides, purine bases, histamine, amino acids, and a diverse proteins and polypeptides. Proteomic analysis of an HLE formerly marketed as Immodin (SevaPharma, CZ) identified 48 unique proteins and polypeptides, which were classified into three functional categories: regulators of immune responses; mediators of inflammation and tissue repair; and proteins involved in cell growth and proliferation [16]. Similarly, LC-MS analysis and peptide sequencing of another HLE preparation, licensed in Mexico under the trade name Transferon Oral, identified monomeric ubiquitin as its predominant peptide component [17]. Various preparations of non-immune human and animal low-molecular-weight leukocyte extracts have demonstrated disease-specific immunomodulatory activity and therapeutic potential in both experimental and clinical settings. Beneficial effects have been reported in viral infections such as HIV and herpes zoster [18,19], pulmonary tuberculosis [20], bacterial sepsis in paediatric patients [21], pathogenic bacteria [22], parasitic infections including *Toxoplasma gondii* [23], cestodes such as *M. vogae* [13,14] and *E. multilocularis* [24]. Moreover, HLEs have been shown to stimulate anti-tumour immunity in experimental breast cancer [25] and prostate cancer [26].

Helminths generally suppress host immunity through secretion of immunomodulatory molecules and extracellular vesicles that interact with multiple immune cell populations, redirecting the immune response away from protective pathways and thereby promoting their own survival [27]. During *E. multilocularis* infection in humans and experimental mice, the initial Th1-dominated response progressively shifts, depending on parasite burden, towards polarised Th2 profile. This transition facilitates parasite establishment while simultaneously promoting tissue repair and limiting excessive inflammatory responses. Concurrently, the parasite induces profound suppression of both innate and adaptive immunity, characterised by expansion of myeloid-derived suppressor cells (MDSCs), alternatively activated (M2) macrophages and Foxp3 ⁺ regulatory T lymphocytes, together with incresed levels of the cytokines IL-10 and TGF-β [9,28,29].

Extracellular vesicles are membrane-bound particles secreted by both eukaryotic cells and by parasites during host infection. These vesicles carry a wide range of bioactive molecules, including microRNAs (miRNAs). miRNAs are small (19–24 nucleotides) non-coding RNAs that regulate gene expression at post-transcriptional level by inhibiting protein translation or destabilising target transcripts [30]. Several worm-derived miRNAs are stably detectable in host serum and have emerge as promising biomarkers for the early diagnosis of parasitic infections. Because these molecules actively modulate host–parasite interactions and contribute to disease pathogenesis and immune regulation system, miRNAs also represent attractive candidates for therapeutic intervention [31,32]. Some parasite-derived miRNAs can interact with endogeneous host miRNA regulatory networks, suggesting that parasites exploit the host’s molecular pathways for their benefits [33]. Guo and Zheng [34] identified seven *E. multilocularis*-specific circulating miRNAs in the serum of infected mice, together with differential expression of 58 host-derived miRNAs. A subsequent study reported that the four most abundant parasite-derived miRNAs (emu-miR-71-5p, emu-let-7-5p, emu-miR-4989-5p/3p, and emu-miR-10-5p) are secreted within extracellular vesicles [35]. However, their contributions to the immunopathogenesis of AE remains poorly understood. Likewise, very little is known about whether albendazole therapy, either alone or in combination with adjunct immunomodulatory compounds, affects circulating levels of parasite-derived miRNAs or how treatment-induced alterations in these miRNAs relate to host immune responses.

The present study investigated the therapeutic efficacy and immunomodulatory effects of HLE, ABZ, and their combination in a murine model of severe subchronic alveolar echinococcosis. Specifically, we evaluated the impact of these treatment regimens on serum levels of selected parasite-derived miRNAs and examined associations with key markers of systemic innate and adaptive immunity, as well as the local immune responses within the parasite-infected peritoneal cavity.

## Materials and methods

### Ethics statement

The animal study was conducted in accordance with guidelines laid down by Government Regulation § 35, no. 377/2012 Coll., establishing requirements for the protection of animals used for scientific purposes or educational purposes as amended by SR Government Regulation No. 199/2019 Coll. All methods are reported per ARRIVE guidelines. The study protocol was approved by the Ethics Committee of the State Veterinary and Food Administration of the Slovak Republic under protocol No. 3920/2022–220.

### Infection and experimental design

Infection with metacestode *E. multilocularis* was maintained in animal facilities under pathogen-free conditions by intraperitoneal passage through gerbils (*Meriones ungulatus*) at the Institute of Parasitology of the Slovak Academy of Sciences. Suspensions of protoscoleces, containing also homogenised cyst´s tissue were prepared from the peritoneal cysts aseptically isolated from the gerbil using Cell dissociation sieve tissue grinder kit in sterile PBS and the concentration of protoscoleces was calculated under the light microscope. The experiment was carried out on 8 weeks old male BALB/c strain of mice from own breeding at the Institute of Parasitology, which were originally purchased from VELAZ (Czech Republic). The animals were housed in a temperature-controlled light cycle room with food and water ad libitum. Mice were infected with dose of approximately 3000 protoscoleces in 0.2ml saline intraperitoneally (i.p.) and were randomly allocated into four groups (n = 6 per group) named as infected (INF), treated with albendazole (ABZ), treated with human leukocyte extract (HLE) and treated with the combination of albendazole and human leukocyte extract (ABZ + HLE). Six uninfected mice served as the healthy control (Ctrl). Treatments were administered daily for 14 consecutive days and started after 4 weeks post-infection (p.i.). After termination of therapy (6 weeks p.i.) mice were subjected to mild anesthesia in a CO_2_ atmosphere and blood was obtained from the retro-orbital plexus for isolation of serum. Mice were then euthanised by cervical dislocation and peritoneal exudate cells (PECs), spleen, liver and peritoneal cysts were collected.

### Treatments and drugs

For oral administration, ABZ (Sigma-Aldrich, St. Louis, MO, USA) was suspended in 1.0% cremophor oil in 0.9% sterile NaCl and stored at 4 °C. Prior administration, aliquots of suspensions were warmed and ABZ was administered orally by gavage at a dose of 15 mg/kg body weight. HLE was delivered intraperitoneally at a dose of 0.2 mL (equivalent to an extract derived from 1 × 10^9^ leukocytes). HLE used in our study was manufactured by Aumed, Ltd. (Prague, Czech Republic), a GMP-certified pharmaceutical company, and supplied exclusively for research purposes. The preparation is registered in the European Union under International Publication No. WO 2021/249581 A1 and patented in the Czech Republic by the Industrial Property Office (Patent No. 34517). According to the manufacturer’s protocol, HLE is a soluble extract of peripheral blood mononuclear cells (PBMCs) obtained from over 150 healthy donors, which were screened at certified transfusion centres and tested negative for HIV 1/2, HCV, HBsAg, and syphilis. The extract contains a complex mixture of proteins and other molecules with a molecular weight below 10 kDa. Each batch was prepared by pooling leukocytes from donors, followed by disintegration using six cycles of freezing at a temperature below −50 °C and thawing at a temperature above +30 °C. The resulting homogenate was concentrated using dialysis to a defined protein concentration, and the low molecular weight fraction was isolated using 10 kDa ultrafiltration membranes and further concentrated by ultrafiltration. The final product was pasteurised and quality-controlled for sterility, endotoxins, and biological activity using proliferation assays on the Jurkat T cell line.

### Efficacy of treatment, cysts weight and histology

Cysts were dissected from the peritoneal cavities of mice, weighted and fixed in 4% paraformaldehyde for histological examination to assess the presence of protoscoleces. The cysts were processed using a standard histological protocol, embedded in paraffin blocks, and sectioned at a thickness of 7 um. Tissue sections were stained with Haematoxylin and eosin stains, both from Sigma Aldrich. (St. Louis, USA) and mounted into Pertex mounting medium (Histolab Products AB, Vastra Frolunda, Sweeden). The liver samples were processed by the same procedures and tissue sections were examined for the presence of cysts. Slides with sectioned cysts and livers were examined using Olympus BX51 microscope (Hachioji, Tokyo, Japan).

### Preparation of splenocytes and peritoneal exudate cells

Spleens were aseptically removed, cut into small pieces and single-cell suspensions were prepared by gentle pressing the tissue between the sterile glass slides in cold complete medium (CM). The CM consisted of RPMI 1640 medium containing stable L-glutamine, supplemented with 10% heat-inactivated foetal calf serum, 100 U/mL of penicillin, (all purchased from Sigma, St. Louis, USA), 100 µg/mL of streptomycin (Biochrom, Berlin, Germany) and 50 µM 2-mercaptoethanol (Merck, Darmstadt, Germany). Unless otherwise stated, all experimental procedures involving cells were performed using this complete medium. Red blood cells were lysed by incubation in cold lysis solution (0.8% NH_4_Cl, 0.84% NaHCO_3_, 0.37% EDTA) for 5 min. Cells were then washed twice with cold PBS, filtered through a 40 µM nylon cell strainers (BD Biosciences, Durham, NC, USA) and counted. ell viability was tested using Trypan Blue exclusion assay. Splenocytes were used for the ex vivo proliferation assay, flow cytometric analysis, and RNA isolation. Peritoneal exudate cells were collected by washing the peritoneal cavities with 5 mL of prewarmed CM, and cells were washed and counted. Aliquots of cell suspensions were used for flow cytometric analysis, ex vivo quantification of NO production and for RNA isolation. Cell suspension intended for RNA isolation were preserved in RNAlater (Sigma) prior freezing at -80°C. Spleen tissue designated for protein isolation was snap-frozen and stored at -80°C until further analysis.

### Flow cytometric analysis

Cells suspensions of spleens and peritoneal exudate cells from individual mice were analysed using flow cytometry to monitor the proportions of lymphoid and myeloid cells subpopulations. Aliquots (0.3 × 10^6^ cells/50 μl) of splenocytes and peritoneal exudate cells were resuspended in FACS buffer and stained with monoclonal anti-mouse antibodies to: CD45.2-PE-Cy7 (clone C.104, eBioscience, San Diego, CA, USA), CD3-FITC (clone: 11–0032) and CD19-PE (clone 1D3, eBioscience). Other cell samples were stained with anti-mouse antibodies to: CD4-FITC (clone GK 1.5) and CD8-PE (clone C:53-6.7); all from eBioscience. NK and NKT cells were detected after staining cells with anti-mouse antibodies to: CD8-PE (clone C:53-6.7) and CD49b-APC (clone DX5), both from eBioscience. Myeloid cell populations were analysed using monoclonal antibodies to: CD11b-FITC (clone M1/70, BioLegend, San Diego, CA, USA), CD68-PE (clone C: FA-11, eBioscience), MHC II-PeCy7 (clone M5/114.15.2, eBioscience), Gr-1-PE (clone C: RB6-8C5) (Biolegend) and its components Ly-6C-Per CP-Cyanine 5.5 (clone HK 1.4) and Ly-6G-PE Cyanine 7 (clone C:16-10A1), both from eBioscience. In addition, eosinophils were detected after staining with antibodies to CD170 SiglecF- PerCPeFluor 170 (clone 1RNM44N, eBiosciences). Cells were incubated for 30 min at room temperature in the dark, washed, and recorded using a FACS Canto analyser (BD Biosciences, Franklin Lakes, NJ, USA). The acquired data were analyzed using the FACS Diva software.

### Cytokine concentrations in serum

Concentrations of cytokines in serum samples from each mouse (n = 6) diluted in PBS (1:4) were examined using ELISA kits for IFN-γ and IL-10, following the instructions (both from Invitrogen, Carlsbad, CA, USA). Cytokines IL-4, IL-17, IL-12 and TGF-β were detected using Kits purchased from Affymetrix-eBioscience, (San Diego, CA, USA). Values were calculated from the calibration curves using standards and are expressed in pg/ml. OD values after stopping reaction with 2N H_2_SO_4_ were measured at 450 nm using the Multiscan FC Plate Reader (Thermo Scientific, Finland). Data are expressed as means ± SD.

### BrdU proliferation assay ex vivo

The proliferation capacity of splenocytes ex vivo was examined using BrdU ELISA cell colorimetric proliferation kit (Roche Diagnostics GmbH, Mannheim, Germany). The suspensions of splenocytes from each mouse/group were diluted to the concentration of 2 × 10^6^ cells/ml in CM (200 μl/well) and incubated in 96-well plates (Corning. Inc., USA) in triplicates for unstimulated and concanavalin A (Con A, 3 μg/ml) stimulated T lymphocytes or lipopolysaccharide stimulated B cells (LPS, 1 μg/ml). Cells were cultured for 72 h at 37 °C, 5% CO_2_ and humidity. BrdU solution was added to the cell suspensions at 5 μM final concentrations 18 h before termination of cultivation. Plates were then centrifuged at 360 *g* for 10 min and after removing the supernatants, plates were dried and further processed following manufacturer´s instructions. Stimulation of cell proliferation was determined as the fold change of absorbance of stimulated versus unstimulated cells for each cell sample. Data represent means ± SEM of technical triplicates (n = 6).

### miRNA isolation and RT-qPCR analysis

For quantification of miRNAs in serum of infected and treated mice, the highly sensitive LNA technology (Qiagen Life Science, Hilden, Germany) was employed. For isolation of microRNAs from serum samples (100 µl/sample) miRNeasy Serum/Plasma Advanced Kit (kat.no 217204) was used. Eluted miRNAs were reverse transcribed using miRNA LNA RT Kit (cat. No. 339340) according to the instructions, except that the final 10 ul volume of cDNA was not diluted 30 times with nuclease-free water. The optimal cDNAs dilution for each analysed miRNA was determined by RT-qPCR reactions using miRCURY LNA SYBR Green PCR Kit (cat.no. 339345) in combination with specific primers sets (miRCURY LNA miRNA PCR Assay). The parasite-secreted miRNAs were analysed using the following specific PCR assays: emu-miR let-7-5p (no. YP02102031), emu-miRNA-71a-5p (no. YP02105281), emu-miR-10a-5p (YP02116163) and emu-miR-4989-5p (YP02119884). For normalisation of Ct values, the mouse-specific miR-16-5p (no. YP00205702), previously identified as stable serum reference miRNA [36] was selected as the endogeneous control. Based on preliminary optimalization experiments, a 1:3 cDNA dilution yielded reproducible amplification and optimal Ct values, therefore all reactions using this dilution were performed. Melting curve analysis was carried out to verify the specificity of amplification and the PCR products were further confirmed by electrophoresis on 4% agarose gels. Ct values were normalized to the miR-16-5p housekeeping gene and relative expression levels were calculated by the 2^-∆∆Ct^ method. Relative expression of parasite secreted miRNAs in treated mice was determined using mean Ct values in infected mice as calibrator. Data represent means ± SEM of technical triplicates.

### NO determination by peritoneal adherent cells ex vivo

The production of nitric oxide (NO) by myeloid peritoneal exudate cells was analysed using Griess reactions. Cell suspensions (1 × 10^6^ cells/ml) were cultured in 24-well plates (Corning, NY, USA) in CM in the presence or absence of lipopolysaccharide (LPS) at the concentration of 1 µg/ml. The plates were incubated for 72 h at 37 °C and 5% CO_2_. Immediately after assay termination, the concentration of NO in the culture supernatants was determined as nitrite (NO_2_^-^). Briefly, 50 µl of a solution containing 1% sulphanilamide and 5% H_3_PO_4_ was incubated with 50 µl supernatants in a 96-well plate for 10 min at room temperature. Subsequently, 50 µl of a second solution (0.1% N-(1-naphthyl) ethylenediamine dihydrochloride) was added to the mixture and the absorbance was measured at 550 nm using the Multiscan FC Plate Reader. Nitrite concentration was calculated from the standard curve of 0.1 M NaNO_2_ dilutions run in parallel with samples and are expressed in nmol/ml. Data represent means ± SEM of technical triplicates.

### RNA isolation from cells and RT-qPCR analysis

Total RNA from the cells was extracted with RiboZol RNA Extraction Reagent (Invitrogen, Carlsbad, CA, USA) after homogenization. The concentration and purity of RNAs were determined using AstraGene spectrophotometer (Harston, Cambridge, UK). After that, 3 μg of total RNA/sample was reverse-transcribed to produce cDNA utilizing RevertAid H Minus M-MuLV Reverse Transcriptase, 100 pmol of oligo dT primers and the reaction included 20 U of RNase inhibitor and 1mM dNTPmix (final) (all from ThermoFisher, Burlington, ON, Canada). The list of genes and corresponding primers including annealing temperature and time is shown in S1 Table (supplementary file). GAPDH (glyceraldehyde-3-phosphate dehydrogenase) was used as the housekeeping control genes. For the qPCR reactions, cDNAs samples served as templates in reactions containing iQ SYBR green master mix (Bio-Rad, Hercules, CA, USA) and gene-specific primer pairs. All reactions were run using CFX96 thermocycler (BioRad, Hercules, CA, USA). Melting curve analysis was carried out to verify the specificity of amplification by confirming the presence of a single product. Ct values were normalized to those of the housekeeping gene and relative gene expression was calculated using the 2^-∆∆Ct^ method with samples from healthy mice serving as the calibration value. Data represent means ± SEM of technical triplicates.

### Isolation of protein nuclear fractions, SDS-PAGE electrophoresis and immunoblot analysis of transcription factors

To quantitatively analyse the expression of nuclear transcription factors, the spleen tissues were homogenized on ice using mortrar and pestle in cell lysis buffer supplemented with a protease inhibitor cocktail. Cytoplasmic and nuclear protein fractions were subsequently isolated using ReadyPrep Protein Extraction Kit (Cytoplasmic/Nuclear) (Bio-Rad, Hercules, CA, USA) according to the manufacturer´s instructions. Proteins concentrations in nuclear fractions were determined in duplicates using Bradford protein assay (Bio-Rad). Equal amounts of nuclear proteins (20 μg/lane) were mixed with 4x mPAGE sample buffer, denaturated at 70°C for 10 min and resolved on 4–20% Bis-Tris gradient precast gels (mPAGE) (Merck) at 140 V using Bis-Tris running bufer. For each sample, electrophoresis was performed in duplicate. Proteins were subsequently transferred onto PVDF membranes (0.45 μm, Merck Millipore, Darmstadt, Germany) using a Mini Trans-Blot Electrophoretic Transfer Cell (Bio-Rad, Hercules, CA, USA) with mPAGE prestained protein ladder included as molecular weight marker. Prior membrane blocking, total protein loading was viusualized after staining with the No-Stain Protein labelling Reagent (Invitrogen, Carlsbad, CA, USA), for 10 min and rinsed with distilled water. Membranes were then blocked in ChemiBlocker (Merck) diluted (1:1) in TBST for 1 h at room temperature. An overnight incubation of membranes with the primary monoclonal anti-mouse antibodies to: Histone H3 (housekeeping- loading control), T-bet, GATA3, Foxp3 and Atf3 (all from Santa Cruz Biotechnology, Dalas, Texas, USA) in blocking solution was performed at 4°C with gentle agitation. After washing with TBST, membranes were incubated for 1 h with gentle agitation in blocking solution containing an HRP-conjugated goat anti-mouse IgG secondary antibody (Abcam, Cambridge, UK). Following additional washes with TBST, protein bands were visualized using the chemiluminescent reagent Clarity Western ECL Substrate (Bio-Rad). Chemiluminescent signals were acquired with the iBright FL1500 Imaging System (Thermo Scientific, Rockford, IL, USA) using Smart Exposure and SmartRange HDR settings. Band intensities were quantified using iBright Analysis Software (version 5.2.2, Thermo Fisher Scientific, Cleveland, OH, USA, RRID:SCR_017632). Signal intensities were first normalized to total amount of loaded protein and subsequently to Histone 3. Two technical replicates were analysed for each protein sample obtained from each mouse (n = 4 per group). Relative protein expression was calculated as the fold change compared with the infected control group and is presented as mean ± SD (n = 8).

### Statistical analyses

Biological analyses were performed in duplicates (protein expression) or triplicates (qPCR, cell proliferation, NO detection) and the mean values were then used to calculate the final mean and standard error of mean (SEM) of technical replicates. Data from flow cytometric analysis and cytokine concentrations in serum represent the mean and standard deviation (SD). Statistically significant differences between the intact, infected and treated groups (indicated by connecting lines) were calculated using either one-way ANOVA followed by Tukey’s multiple comparison test or two-way ANOVA, followed by Tukey´s multiple comparison test. Normal gaussian distribution of data was verified using Shapiro-Wilk test and adequate power of ANOVA test indicated F values and α = 0.05. Data were evaluated with GraphPad Prism (version 11.0.2.) (GraphPad Software, Inc., San Diego, CA, USA) and statistical differences in Figures are denoted as * p < 0.05, ** p < 0.01 and *** p < 0.001.

## Results

### HLE enhanced albendazole-mediated reduction of cyst growth in vivo

**Fig 1:**
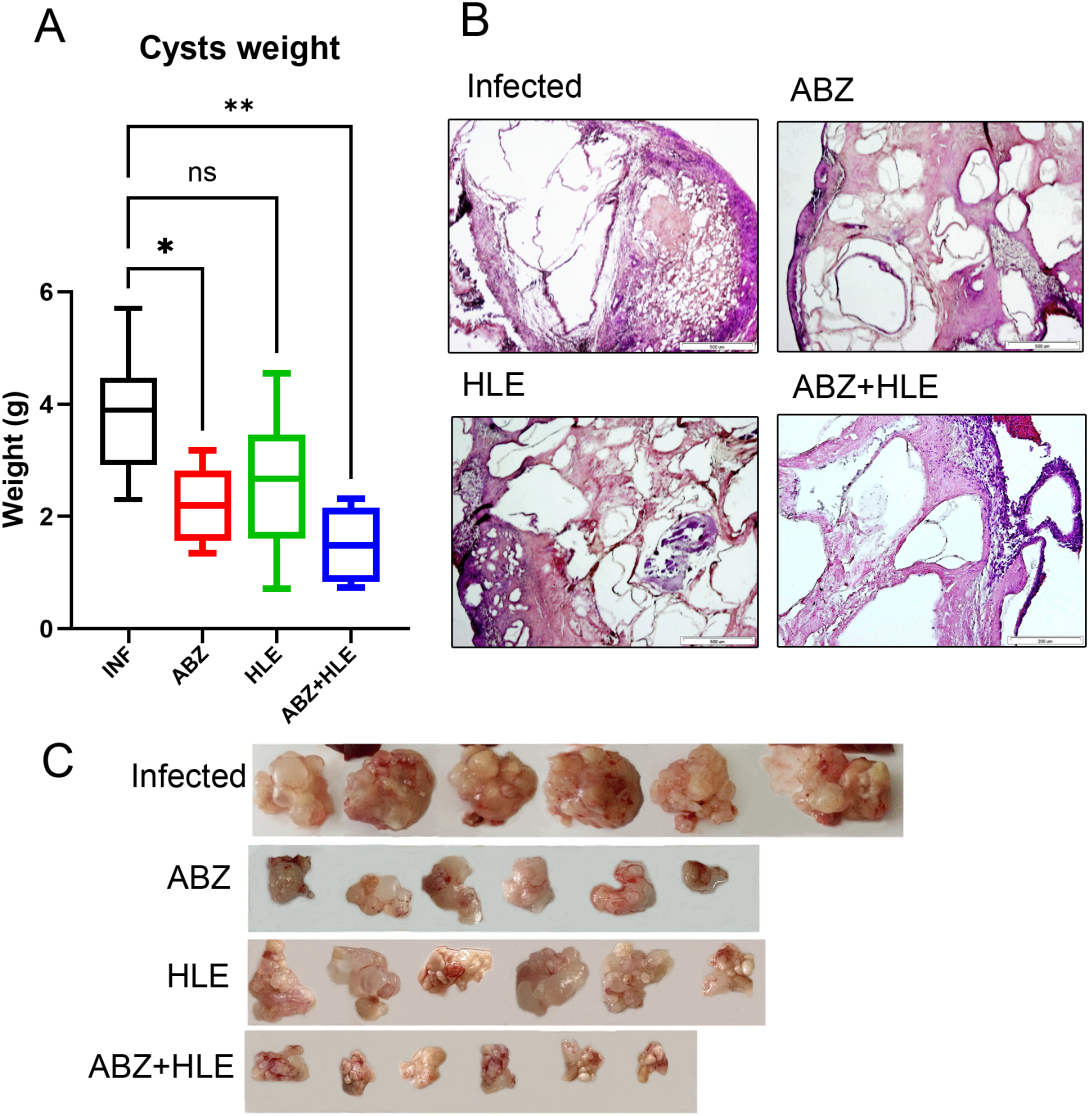
A. the effects of therapies on parasite cysts weight, * p < 0.05, ** p < 0.01. B, representative immages of the cysts´s histological sections from all groups and protoscleces were detected. Scale bar = 200µm. C, gross images of cysts from individual groups of mice.

We previously demonstrated that human low-molecular-weight leukocyte extract (HLE), administered to mice significantly enhanced the therapeutic efficacy of albendazole (ABZ) in mice infected with the asexually proliferating larval stages of *Mesocestoides vogae*, resulting in a greater reduction of parasite burden in both the peritoneal cavity and liver [14,15]. In the present study, we evaluated whether HLE exerts a similar adjuvant effect during experimental *Echinococcus multilocularis* infection. Mice were treated during the subchronic phase of infection, beginning at week 4 post-infection (p.i.), with oral ABZ, intraperitoneal HLE, or a combination of both for 14 consecutive days. All mice developed peritoneal metacestode infection, although cyst mass varied among individual mice within each experimental group. ABZ treatment significantly reduced cyst mass compared with untreated controls (p < 0.05; Fig 1A and 1C). HLE monotherapy produced a moderate reduction in parasite burden, whereas the combination of HLE and ABZ resulted in most pronounced decrease in cyst weight (p < 0.01) (Fig 1C). Histological examination of paraffin-embedded cyst sections revealed no detectable protoscoleces in cysts from any of the experimental groups (Fig 1B).

### HLE alone and in combination with ABZ suppressed circulating *E. multilocularis*-derived miRNA

**Fig 2:**
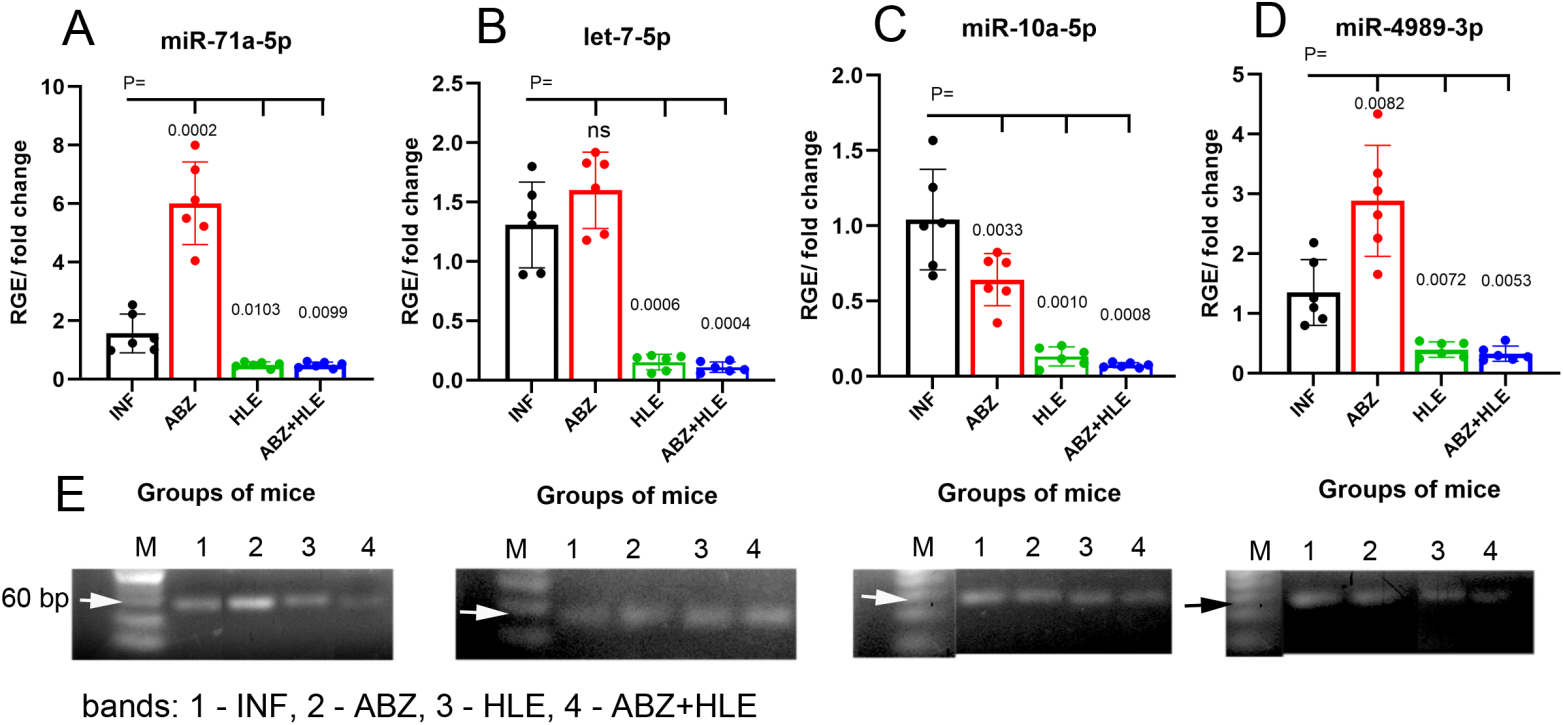
Serum levels of miRNAs: A, miR-71a-5p; B, let-7-5p; C, miR10a-5p and D, miR-4989-3P in infected untreated and mice treated with ABZ, HLE and ABZ + HLE measured as relative gene expression after termination of drugs administration. Bellow figures are electrophoretic profiles of miRNAs from quantitative RT-PCR (P values are shown above individual bars). Data represent the means ± SEM (mean of technical triplicates, n = 6).

An increasing number of studies demonstrate that parasite-derived microRNAs (miRNAs) circulate stably within the host’s body fluids, including serum, where they have emerged as promising biomarkers of helminth infection [31,37]. To assess whether treatment-induced reductions in cyst mass were associated with changes in circulating parasite-derived miRNAs, we quantified the four the most abundant *E. multilocularis* miRNAs: emu-miR-71a-5p, emu-let-7-5p, emu-miR-10a-5p and emu-miR-4989-3p [34,35], using LNA-based RT-qPCR. Murine miR-16-5p, which is stably expressed in serum mice, served as the endogenous reference. As shown in Fig 2A–2D, ABZ treatment was associated with increased levels of emu-miR-71a-5p, emu-let-7-5p and emu-miR-4989-3p, whereas only emu-miR-10a-5p was significantly reduced compared with untreated infected controls (p = 0.0033). In contrast, all four parasite-derived miRNAs were significantly reduced in mice treated with HLE alone or in combination with ABZ (p < 0.001). In mice receiving combination therapy, the decline in circulating miRNA levels correlated significantly with the reduction in metacestode weight.

### HLE and combination therapy promoted Th1/Th2 – cytokine responses while suppressing regulatory cytokines in serum

**Fig 3:**
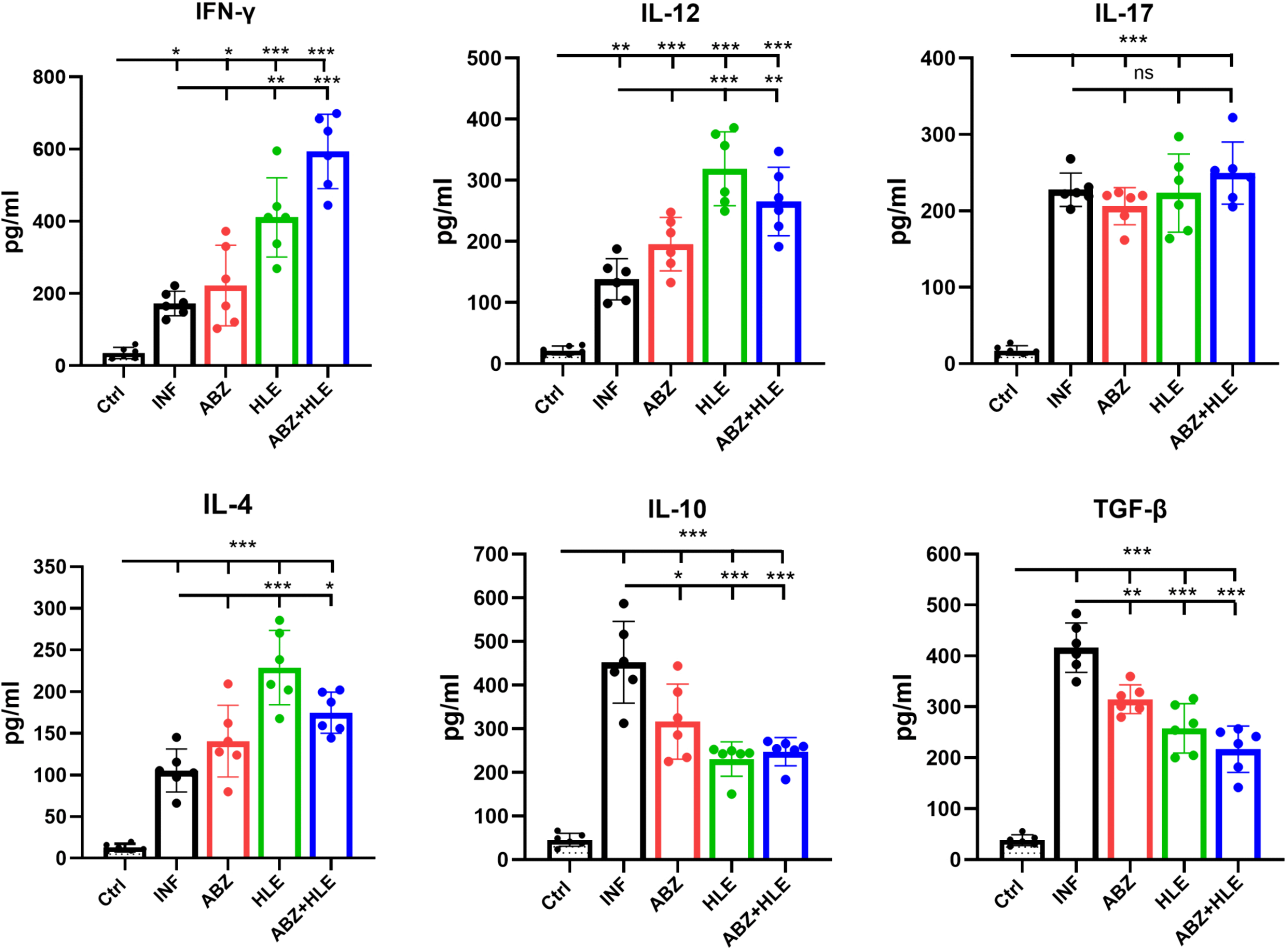
Concentrations of cytokines IFN-γ, IL-12, IL-17, IL-4, IL-10, IL-4 and TGF-β, determined.

Parasite- derived miRNAs transported in extracellular vesicles have been implicated in the modulation the functions of host immune cells through regulation of genes involved in immunopathology. We therefore examined whether treatment-induced changes in circulating parasite-derived miRNAs were accompanied by alterations in systemic cytokine responses. Serum concentrations of representative Th1 (IFN-γ, IL-12), Th2 (IL-4), Th17 (IL-17) and T regulatory (TGF-β, IL-10) cytokines were determined (Fig 3). ABZ monotherapy did not significantly affect Th1, Th2 or IL-17 cytokine levels but significantly reduced TGF-β (p < 0.01) and IL-10 (p < 0.001). In contrast, HLE administration significantly elevated the production of IFN-γ, IL-12 and IL-4 cytokines, whereas IL-17 levels remained unchanged. A comparable cytokine profile was observed following combination therapy. Furthermore, HLE alone and in combination with ABZ significantly reduced the T regulatory-associated cytokines TGF-β and IL-10 (p < 0.001).

with ELISA assays in the serum from control healthy mice (Ctrl), infected mice (INF), and infected mice treated with albendazole (ABZ), HLE, and their combination (ABZ + HLE). Data represent the means concentration ± SD (n = 6). Significantly different values between selected groups of mice are indicated by connecting lines as * *p* < 0.05, ** *p* < 0.01 and *** *p* < 0.001.

### Combination therapy upregulated Th1 and downregulated Th2/Treg lineage- specific transcription factors

**Fig 4:**
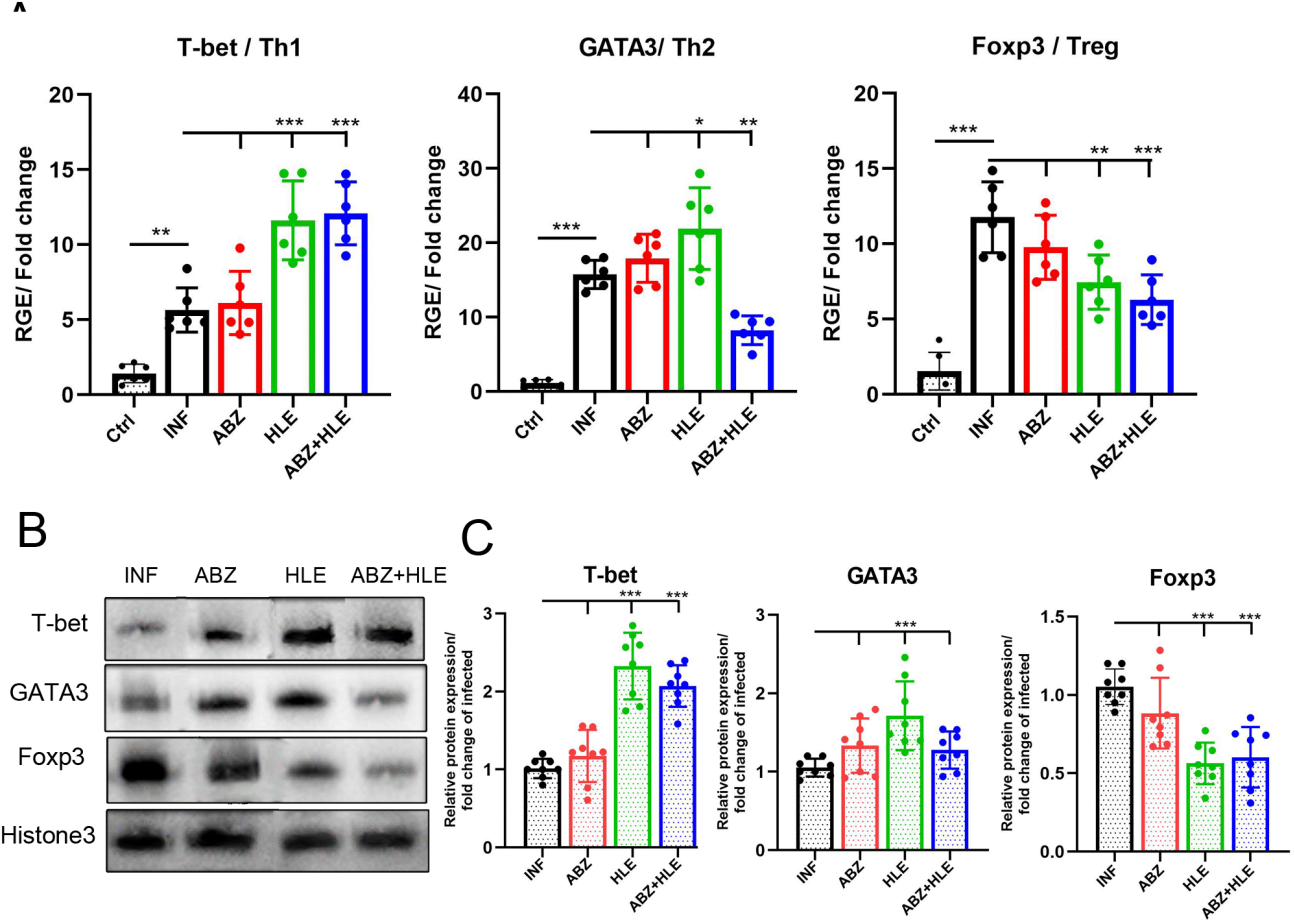
A. relative gene expression of the key transcription factors defining Th1, Th2 and Treg (*T-bet, GATA3, and Foxp3* genes) responses in splenocytes isolated from individual groups of mice, assessed by qPCR. Data are shown as means ± SEM (means of technical triplicates) (n = 6); B, representative immages of immunoblot analysis of protein expression of T-bet, GATA3, and Foxp3 transcription factors and C, relative protein expression expressed as fold change of infected group. Samples of nuclear proteins from 4 mice/group were analysed in duplicates and data are expressed as means ± SD (n = 8). Significat differences between groups are indicated by conneting lines as * p < 0.05 ** p < 0.01, *** p < 0.001.

To determine whether the treatment-induced changes in serum cytokine profiles were accompanied by alterations in T-helper cell lineage commitment, we next examined the expression of lineage-defining transcription factors in splenocytes: *T-bet* (Th1), *GATA3* (Th2), and *Foxp3* (Tregs). *T-bet* drives Th1 differentiation and IFN-γ production [38], *GATA3* promotes Th2 differentiation while suppressing Th1 polarisation [39], and *Foxp3* is master transcription factor required for regulatory T-cell development and function [40]. ABZ-treated mice exhibited *T-bet* and *GATA3* m-RNA expression levels comparable to those of infected untreated mice, whereas Foxp3 mRNA levels were significantly reduced (Fig 4A and 4B). HLE treatment significantly upregulated *T-bet* (p < 0.001) and *GATA3* (p < 0.05) expression, while reducing *Foxp3* m-RNA levels (p < 0.05), consistent with observed serum cytokine profiles. Combination therapy induced the highest *T-bet* expression (p < 0.001) accompanied by the highest serum IFN-γ levels, while significantly reducing both *GATA3* (p < 0.01) and *Foxp3* (p < 0.01) expression. These transcriptional changes were confirmed by immunoblot analysis of transcription factors shown in Fig 4B and 4C.

### All treatment regimes reduced infection-induced expression of Breg -associated transcription factor but not CD9 surface marker

**Fig 5:**
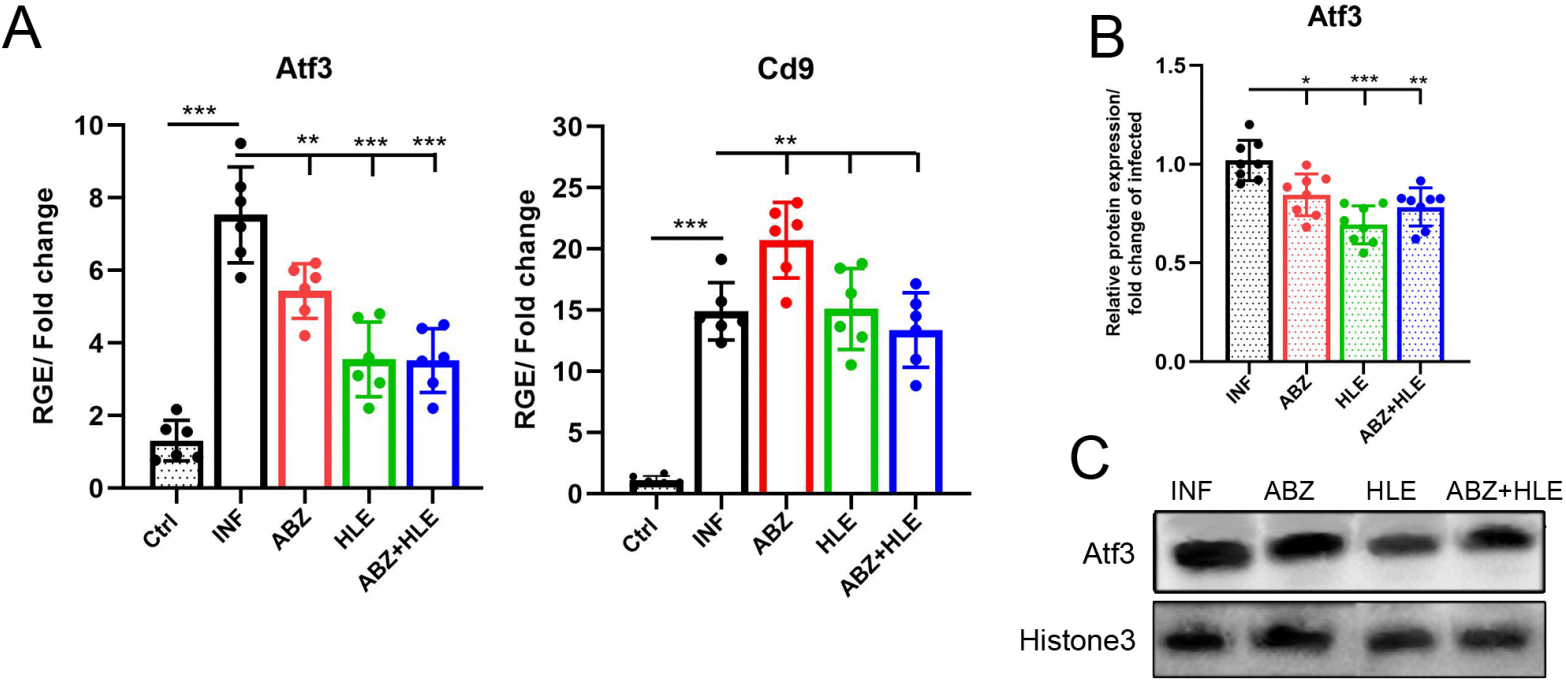
Gene expression of B regulatory cells associated markers Cd9 and Atf3 in splenocytes from individual groups of mice following therapies. Relative mRNA expression levels of Cd9 and Atf3 were assessed by qPCR. Data are shown as means ± SEM (means of technical triplicates) (n = 6). B, relative Atf3 protein expression expressed as fold change of infected group, C, representative immages of immunoblot analysis of Atf3 transcription factor. Samples of nuclear proteins from 4 mice/group were analysed in duplicates and data are expressed as means ± SD (n = 8) Significat differences between groups are indicated by conneting lines as * p < 0.05 ** p < 0.01, *** p < 0.001.

To assess modulation of suppressive B-regulatory cell (Breg) responses, we measured splenic mRNA expression of the Breg-associated surface marker *CD9* and the Breg-intrinsic transcription factor *Atf3* [41]. Compared with intact controls, *CD9* expression was significantly upregulated in all infected groups and further elevated following ABZ therapy (p < 0.01). HLE and combination treatment did not significantly alter CD9 expression (Fig 5A). In contrast, the highly elevated *Atf3* gene expression observed in untreated infected mice (Fig 5A; p < 0.001) was significantly reduced in all treated groups, most notably in the ABZ + HLE -treated group (p < 0.01). These transcriptional findings were corroborated by Atf3 protein expression determined by immunoblot analysis (Fig 5B and 5C). Collectively, strong downregulation of suppressive T- and B-cell pathways after HLE and combination therapy aligns with the observed reduction in infection burden.

### HLE monotherapy and in combination with albendazole, restored impaired T- and B-cell proliferative responses

**Fig 6:**
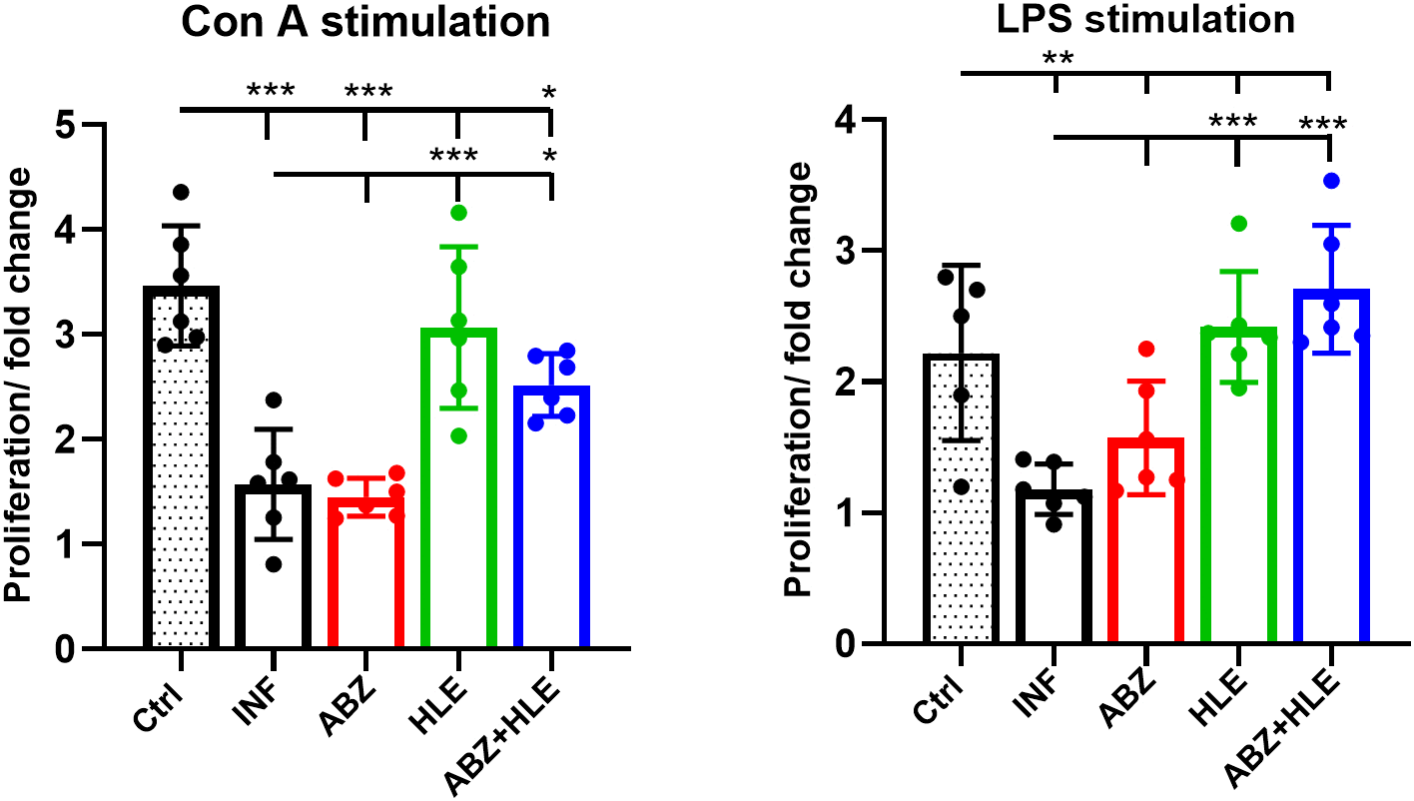
Proliferative responses of of T and B lymphocytes from spleesn of mice after mitogenic stimulation determined by BrdU incorporation assay after 72 h culture. Data represent fold change of proliferation after ConA stimulation (T cells) and after LPS stimulation (B cells). Data are means ± SEM (means of technical triplicates) (n = 6); * p < 0.05 ** p < 0.01, *** p < 0.001.

To assess wheather treatment affected lymphocyte function independently of T-helper cell polarisation, we evaluated proliferative responses of splenocytes to Con A (T cell mitogen) and LPS (B cell mitogen) using BrdU incorporation after 70 h of culture ex vivo. Proliferative responses are expressed as fold change relative to unstimulated cells (Fig 6).

Splenocytes isolated from infected untreated and ABZ-treated mice showed markedly reduced Con A-induced T-cell responsiveness compared with healthy controls (p < 0.001). HLE treatment restored T-cell proliferative response to levels comparable to those of healthy mice, whereas combination therapy produced a partial but significant recovery (p < 0.05). LPS-induced B-cell proliferation was lower in infected mice than in healthy controls (p < 0.01). ABZ monotherapy produced only a modest improvement, whereas HLE alone and in combination with ABZ significantly restored or enhanced LPS-induced B-cell proliferation (p < 0.001).

### HLE monotherapy or in combination with albendazole, reduced splenic macrophage populations with impaired antigen-presenting capacity

To characterise the infection- and treatment-induced alterations in splenic immune cell populations, we performed flow cytometric analysis of the major myeloid and lymphoid subsets. Representative cytospin preparations of splenocytes from infected mice are shown in Fig 9A. Myeloid cell populations were identified using antibodies against CD11b, CD68, MHCII, Gr-1 and its subsets Ly6C and Ly6G. CD11b is broadly expressed by myeloid cells, whereas CD68 is a well-established marker of macrophages and monocytes.

**Fig 7:**
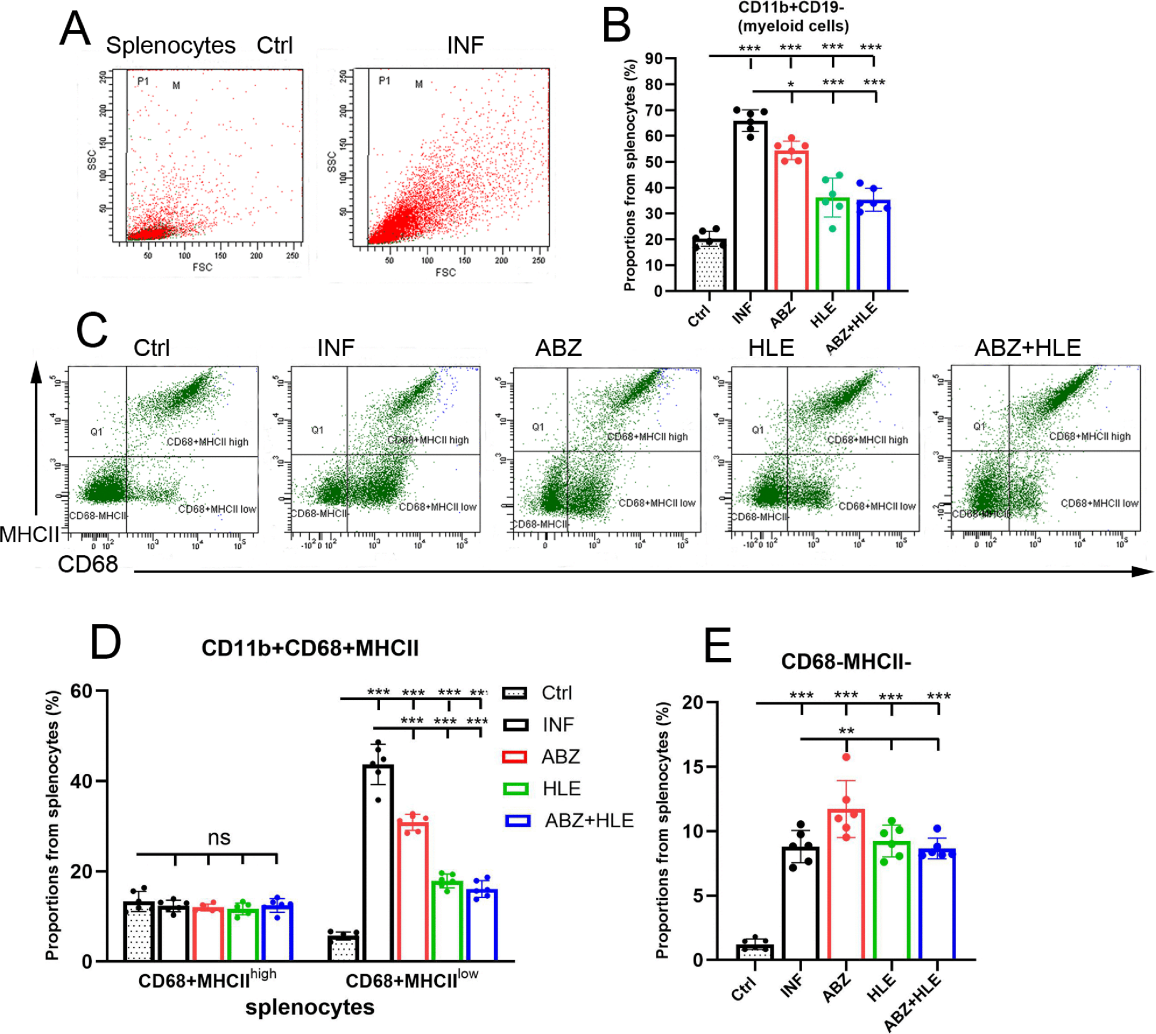
Phenotypic analysis of selected myeloid populations in spleens from individual groups of mice analysed by flow cytometry. A, representative dot plots showing differential profile of splenocytes from healthy control and infected mice. B, the proportions of CD11b^+^CD19^-^ myeloid cells in the whole populations of splenocytes. C, representative dot plots and D, the proportion of CD68 + MHCII^+^ macrophages in all groups. E, the proportions on CD68-MHCII- granulocytes. Data are means ± SD (n = 6); * p < 0.05 ** p < 0.01, *** p < 0.001.

**Fig 8:**
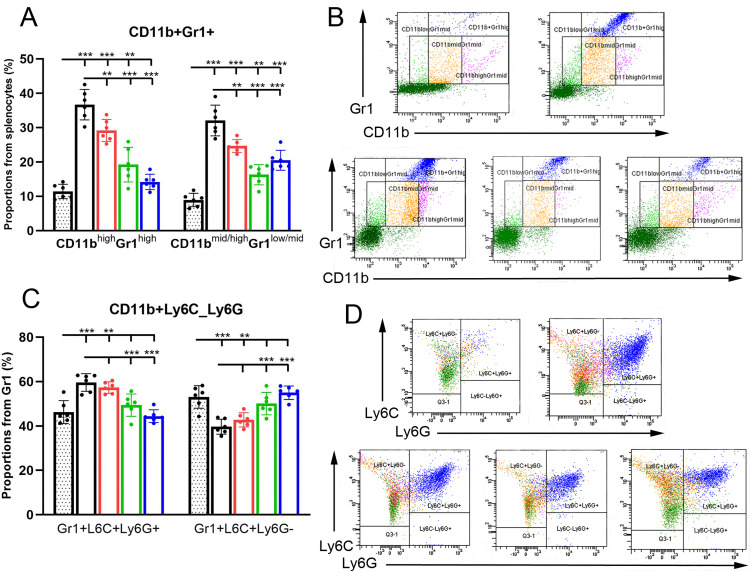
Phenotypic analysis of selected myeloid populations in spleens from individual gropus analysed by flow cytometry. A, the proportions of CD11b^+^Gr-1^+^ myeloid-derived suppresor cells within populations of splenocytes and B, the representative dot plots of cells from all groups of mice. C, the proportions and D, representative dot plots of CD11b^+^Ly6C-Ly6G myeloid suppressor cells gated on CD11b^+^Gr-1^+^ from all groups of mice. Data are means ± SD (n = 6); ** p < 0.01, *** p < 0.001.

Consistent with infection progression spleens of *E. multilocularis* infected mice exhibited pronounced infiltration of CD11b⁺ myeloid cells comprising both monocytic and granulocytic populations (Fig 7A). These cells represented 66.01 ± 4.1% of total splenocytes, compared with 20.93 ± 2.4% in healthy controls (Fig 7B). All therapeutic intervention reduced the proportions of total CD11b^+^ myeloid cells, with the greatest decline observed in mice following HLE monotherapy (36.21 ± 7.5%) and in combination with albendazole (35.42 ± 4.4%, both p < 0.001).

Given the previously reported impaired macrophage antigen presentation resulting from reduced MHC II expression during *E. multilocularis* infection [42], we next analysed the proportions of CD11b⁺CD68 ⁺ macrophages expressing either high or low levels of MHC II (Fig 7C and 7D). Despite of substantial differences in the abundance of total myeloid cells among groups, the proportions of CD68 ⁺ MHC II^high^ macrophages remained comparable to that observed in healthy mice. In contrast, macrophage subset with low MHC II expression, which accounted for only a minor subset in spleens from healthy mice (13.31 ± 2.2%), was markedly expanded in infected mice (43.70 ± 4.7%). All treatment regimes reduced proportions of these macrophages, with the strongest effects observed following HLE therapy (p < 0.001) and combination therapy (16.10 ± 1.8%; p < 0.001).

**Fig 9:**
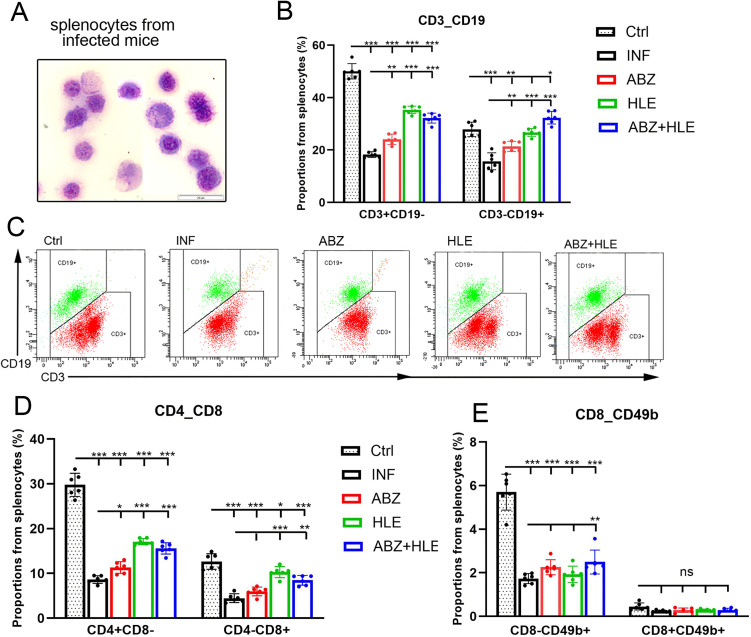
Phenotypic analysis of lymphoid spleen cell populations isolated from control healthy mice (Ctrl), infected mice (INF), and infected mice treated with albendazole, HLE, and their combination (ABZ + HLE) using flow cytometry. A, the representative cytospin preparation of splenocytes from infected mice showing the mixed lymphoid and myeloid populations. B, the proportions of T and B lymphocytes and C, representative dot plots of CD3- CD19 cells from all groups. D, the proportions of CD4 ^+^ Th lymphocytes and CD8 ^+^ Tc lymphocytes, E, the proportions of CD8^-^CD49b^+^NK cells and CD8 ^+^ NK49 ^+^ NKT cells within splenocytes. Data are means ± SD (n = 6); ** p < 0.01, *** p < 0.001.

Granulocytes, defined as CD11b⁺CD68 ^−^ MHC II^−^ cells, were readily identified in splenic cytospin preparations (Fig 9A) and represented 8.80 ± 1.2% of splenocytes in infected mice (Fig 7E). Their proportions increased significantly following albendazole monotherapy (11.72 ± 2.2%; p < 0.01), whereas HLE alone or in combination did not substantially alter granulocyte proportions.

### HLE administration attenuated the infection - induced expansion of splenic myeloid-derived suppressor cells

To further chcracterize the effects of treatment on immunosuppressive myeloid populations, we analysed splenic myeloid-derived suppressor cells (MDSCs) which are phenotypically defined by the co-expression of CD11b and Gr-1 and the intensity of Gr-1 expression reflects distinct maturation stages [43]. Flow cytometric analysis identified several distinct CD11b⁺Gr-1 ⁺ subpopulations differing in the intensity of Gr-1 expression (Fig 8A and 8B). The CD11b^high^Gr-1^high^ population was substantially elevated in infected mice (36.68 ± 4.4%), and was significantly reduced following albendazole monotherapy (p < 0.01), HLE monotherapy (p < 0.001) and most prominently following combination therapy (14.20 ± 2.2%, p < 0.001). Similarly, three additional CD11b^mid/high^ Gr-1^low/mid^ populations were significantly increased in infected mice (32.09 ± 4.1%) and were reduced by all therapies, with the most pronounced decline observed in mice treated with HLE alone (16.29 ± 2.9%, p < 0.001).

To further characterise Gr-1 ⁺ myeloid subsets, we analysed Ly6C and Ly6G co-expression. Gr-1 is a complex antigen composed of Ly6G which is present in healthy mice primarily on mature granulocytes and Ly6C on immature monocytes/macrophages. Two predominant subsets were detected: Gr-1 ⁺ Ly6C⁺Ly6G⁺ and Gr-1 ⁺ Ly6C⁺Ly6G^−^ (Fig 8C and 8D). A minor Ly6C^−^Ly6G⁺ population (<2% of Gr-1 cells) was excluded from further analysis. The Ly6C⁺Ly6G⁺ population represented 46.28 ± 5.1% of Gr-1 ⁺ cells in healthy mice, and was significantly increased in infected mice (59.58 ± 3.9%, p < 0.001). A similarly increased proportion was observed following ABZ monotherapy (p < 0.01). In contrast, HLE therapy either alone or in combination with albendazole restored this population to values comparable to those of healthy controls (44.43 ± 2.8%). Conversely, the monocytic Ly6C⁺Ly6G^−^ subset was markedly reduced in infected mice (39.66 ± 4.3%, p < 0.001) compared with healthy controls. Treatment with HLE alone and the combination therapy significantly increased this subset (both p < 0.001), restoring it to levels approaching those observed in healthy mice (55.01 ± 3.0%). Together, these data support the conclusion that *E. multilocularis* infection induces a strong influx of MDSCs with reduced MHC II expression and elevated Gr-1 levels across multiple maturation stages. HLE markedly diminished these suppressive myeloid subsets, thereby enhancing the antiparasitic effects of albendazole.

### Individual therapies differentially modulated splenic lymphoid cell populations

The infection-induced expansion of splenic myeloid cells was accompanied by a marked decline in the proportions of lymphoid cells. Representative cytospin preparation of splenocytes from infected mice, illustrating the mixed lymphoid and myeloid infiltrate, is shown in Fig 9A. Flow cytometric analysis (Fig 9B and 9C) demonstrated that CD3 ⁺ T cells represented the smallest lymphoid subset in infected mice (18.25 ± 1.1%), compared with healthy controls (50.06 ± 2.9%). Albendazole therapy induced only a modest increase in CD3 + T cell proportions, whereas HLE alone and in combination with ABZ significantly restored this population (p < 0.001), consistent with the enhanced proliferative responses shown in Fig 6A.

The proportions of CD19 + B cells, which were lower than that of T cells in healthy mice, declined further during infection (p < 0.001). All treatments significantly restored B cell proportions, with the greatest restoration observed following combination therapy (32.32 ± 2.4%), exceeding values in healthy mice (27.86 ± 2.7%, p < 0.001). This finding paralleled the enhanced LPS-induced proliferative responses shown in Fig 6B.

Therapy-induced changes in total CD3 ⁺ T-cell proportions were reflected in CD4 ⁺ T helper cell frequencies (Fig 9D), with the most pronounced increase observed following HLE treatment. Cytotoxic CD8 ⁺ T cells were markedly reduced during infection (4.46 ± 0.9% vs. 12.64 ± 1.7% in healthy controls, p < 0.001). All treatments increased CD8 ⁺ T cell abundance, with the strongest effect seen in the HLE-treated group (10.28 ± 1.2%, p < 0.001) and combination therapy.

Natural killer (NK) cells were previously reported to decline during *E. multilocularis* infection alongside with reduced IFN-γ production [44]. These cells were significantly reduced in infected mice (p < 0.001) (Fig 9E). Combination therapy partially restored the proportions of NK cells (p < 0.01), whereas HLE or ABZ monotherapy produced less pronounced effects. NKT cells (CD8 ⁺ CD49b⁺), which represented a only a minor lymphocyte subset in all groups, was not significantly modulated by therapies. Collectively, these findings demonstrate substantial restoration of T and B lymphocyte populations following HLE based therapy, accompanied by reduced myelopoiesis of suppressive phenotypes.

### HLE and combination therapy most effectively reduced peritoneal myeloid cells and elevated T- and B- lymphocyte abundance

**Fig 10:**
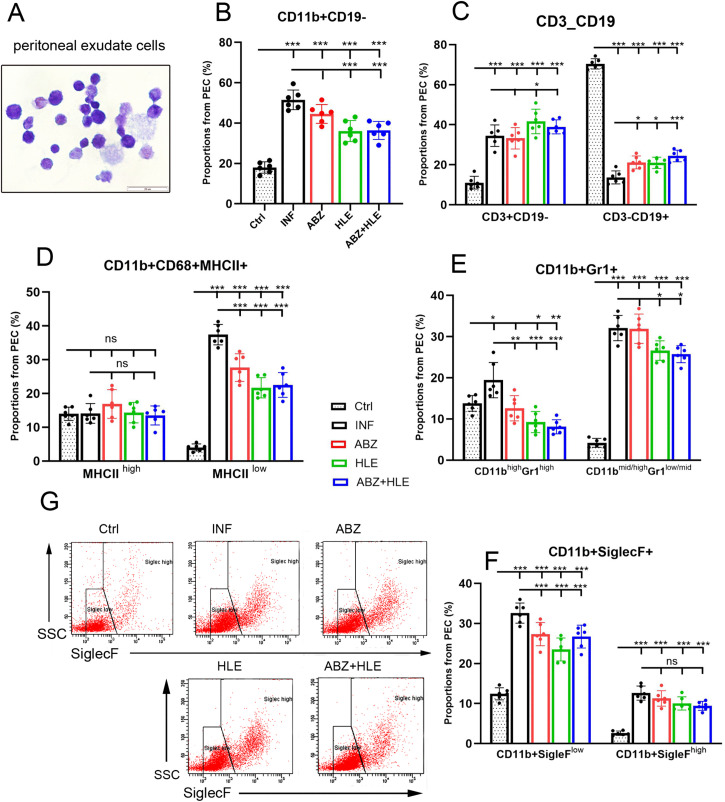
Phenotypic analysis of individual peritoneal cell populations performed by flow cytometry. A, the representative cytospin preparation of peritoneal exudate cells from infected mice. B, the proportions of CD11b+CD19- myeloid derived cells in control, infected untreated mice and treated with ABZ, HLE and ABZ + HLE after termination of therapies. C, the proportions of CD3 T lymphocytes and CD19 B lymphocytes. D, the proportions of CD11b^+^CD68 ^+^ MHC II^+^ macrophages, E, the proportions of CD11b^+^Gr-1 ^+^ myeloid derived suppressor cells. G, representative dot plots of CD11b^+^SiglecF^+^ eosinophils from all groups of mice and F, the proportions of two populations of eosinophils. Data are shown as means ± SD (n = 6); * p < 0.05 ** p < 0.01, *** p < 0.001, ns-not significant.

In our experimental model, intraperitoneal inoculation with approximately 3,000 protoscoleces resulted exclusively in the development of cysts within the peritoneal cavity. The progressive parasite growth induced a pronounced inflammatory response, characterised by the accumulation of large numbers of immune cells with mixed monocytic and granulocytic phenotypes, as demonstrated on cytospin preparations of peritoneal exudate cells (PECs) (Fig 10A). Flow cytometric analysis of PECs showed a marked expansion of CD11b⁺CD19 ^−^ myeloid cells in infected mice (51.52 ± 4.8%), compared with healthy controls (17.08 ± 2.8%) (Fig 10B). All treatments reduced the proportions of these cells, with the most pronounced decline observed following HLE monotherapy (36.05 ± 5.2%) and combination therapy (35.35 ± 4.7%) (both p < 0.001).

The reduction in myeloid populations was mirrored by a corresponding increase in lymphoid cells, which accounted for approximately 45% of total PEC in infected, untreated mice. Flow cytometric analysis of CD3 ⁺ T cells and CD19 ⁺ B cells gated on total PEC is shown in Fig 10C. In contrast to the reduced T cell counts observed in spleens of infected untreated mice, peritoneal T-cell populations were significantly elevated compared with healthy controls (p < 0.001). HLE monotherapy produced a modest but significant further increase (p < 0.05), whereas ABZ monotherapy had no significant effect. CD19 + B cells represented the dominant lymphocyte population in the peritoneal cavity of healthy mice (70.41 ± 2.5%). Infection resulted in a profound reduction in B-cell numbers (13.71 ± 3.2%), whereas all treatments significantly increased their proportions, with the strongest restoration observed following combination therapy (24.40 ± 2.8%, p < 0.001).

### HLE and combination therapy reduced peritoneal myeloid cells with suppressive phenotypes

We next investigated whether the therapies modulated local myeloid populations in the peritoneal cavity in a manner comparable to that observed in the spleens. Peritoneal macrophages, identified by expression of CD11b, CD68, and MHC II accounted for approximately 95% of the total myeloid cells. Mature resident macrophages expressing high levels of MHC II represented only 14.09 ± 2.9% of total PECs in infected mice, and none of the therapies significantly affected this population (Fig 10D). In contrast, infection was associated with a marked expansion of immature CD11b+CD68 + MHC II ^low^macrophages (M2 type) (37.42 ± 3.0%). All therapies significantly reduced the proportion of this population, with the greatest reductions observed following HLE monotherapy (21.70 ± 3.0%) and the combination therapy (22.51 ± 3.6%).

To further characterize suppressive myeloid populations, Gr-1 expression was analysed to identify DSC-like cells (Fig 10E). CD11b^high^ Gr-1^high^ subset represented 19.44 ± 4.2% of total PECs in infected mice and was reduced following albendazole therapy (p < 0.01), with even greater reductions observed after HLE and the combination therapy (p < 0.001). This population was clearly distinguishable from several smaller populations detected as CD11b^mid/high^ Gr-1^low/mid^, which accounted for 32.07 ± 3.0% of PECs in infected mice. HLE alone and in combination with albendazole significantly reduced abundance of these populations (p < 0.05).

We additionally evaluated the proportions of CD11b⁺ granulocytes expressing SiglecF, a canonical eosinophil marker [45], while taking into account that low SiglecF expression has also been reported in immature myelomonocytic cells. Representative dot plots from all experimental groups are shown in Fig 10F. CD11b⁺SiglecF^low^ cells represented 12.42 ± 2.6% of total PECs in healthy mice and were markedly elevated during infection (32.52 ± 2.5%, p < 0.001) (Fig 10G). All therapies significantly reduced this population, with the greatest reduction induced by HLE monotherapy (23.53 ± 2.8%). In contrast, mature eosinophils (CD11b⁺SiglecF^high^) formed a relatively small population in all groups. Although their numbers increased significantly in infected mice (p < 0.001), treatment-induced reductions were modest.

### HLE and combination therapy, but not albendazole, reduced nitric oxide production by LPS-stimulated peritoneal macrophages ex vivo

**Fig 11:**
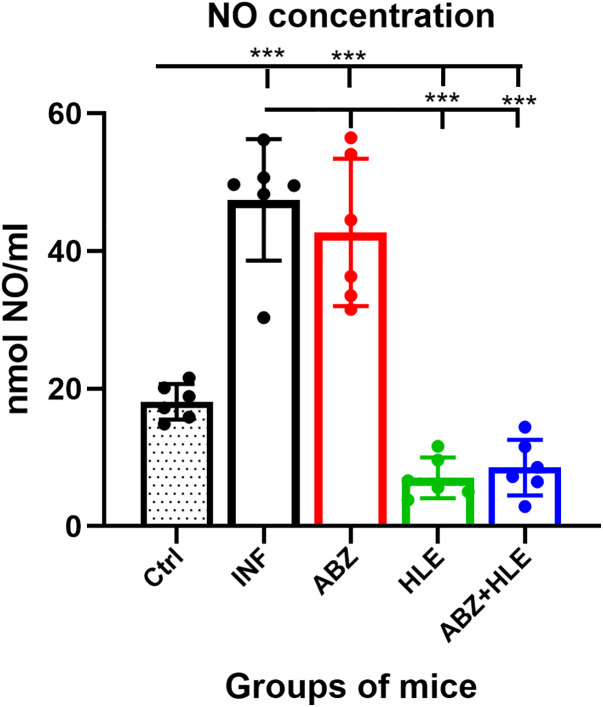
The concentration of NO determined in the culture supernatants of LPS-stimulated peritoneal exudate cells (1x10^6^) from control, infected untreated and infected mice treated with ABZ, HLE and ABZ + HLE ex vivo. NO was determined as nitrite (NO_2_) using Griess reagent. Data are shown as mean ± SEM (means of technical triplicates) (n = 6); *** p < 0.001.

Nitric oxide (NO) plays a central role in immunosuppression in *E. multilocularis* infection [46], and its elevated production has been associated with profound suppression of splenic lymphocyte proliferation. To evaluate the effects of treatment on NO production by peritoneal exudate cells, NO concentration was measured in culture supernatants following ex vivo stimulation with LPS (Fig 11). PECs were cultured for 72 h, and NO production was quantified as an indicator of inducible nitric oxide synthase (iNOS) activity. LPS-stimulated PECs from infected untreated mice produced significantly higher levels of NO than those from healthy controls (p < 0.001). Similarly, elevated NO production was observed following ABZ monotherapy. In contrast, HLE treatment, either alone or in combination with albendazole, significantly reduced NO production compared with untreated infected mice (p < 0.001).

### HLE and combination therapy, but not ABZ attenuated Th1/Th2/Treg cytokine responses in the peritoneal cavity

**Fig 12:**
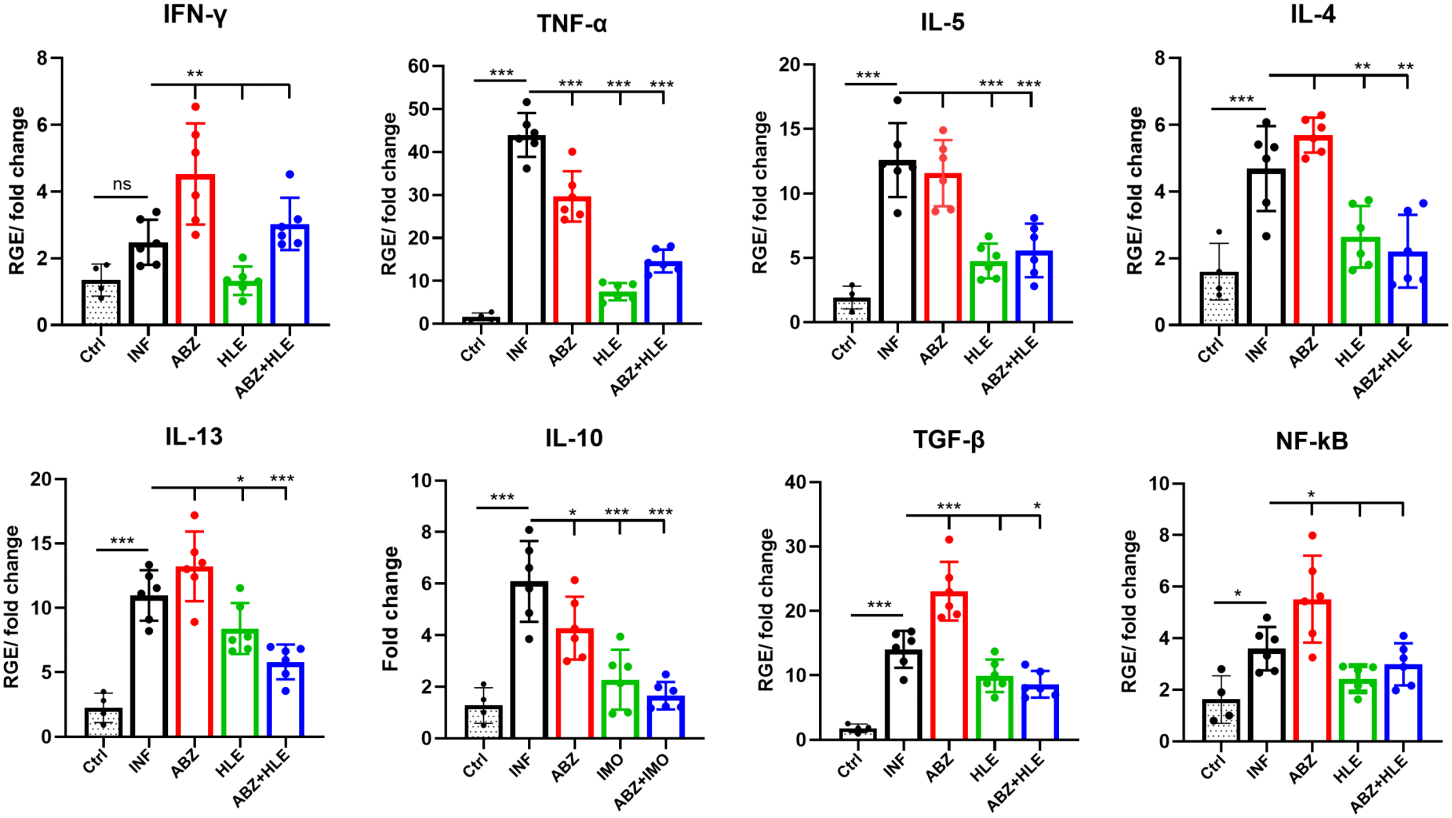
Relative gene expression of mRNAs for selected cytokines and nuclear factor NF-κB in peritoneal exudate cells isolated from control, untreated infected mice and infected treated with ABZ, HLE and ABZ + HLE. Relative gene expression was calculated via the 2^−ΔΔCt^ method, using data for control healthy mice as the calibrator. Data are shown as mean ± SEM (means of technical triplicates) (n = 6); * p < 0.05 ** p < 0.01, *** p < 0.001.

To characterize local immune responses following treatment, cytokine mRNA expression was analysed in PECs (Fig 12). Compared with healthy mice, PECs from infected mice displayed only moderately elevated IFN-γ transcript levels and significantly increased expression of Th1 (TNF-α), Th2, and T regulatory cytokine genes. Albendazole therapy markedly stimulated mRNA expression of IFN-γ, TNF-α, IL-5, IL-4, IL-13, TGF-β, and the transcription factor NF-κB (all p < 0.01) in PECs. HLE treatment resulted in a markedly different profile and markedly reduced the expression of IL-10, TGF-β, IL-5 and TNF-α (all p < 0.001), while producing moderate reductions in IFN-γ, IL-4, IL-13, and NF-κB expression. Combination therapy induced a broadly similar transcriptional profile, although IFN-γ expression remained moderately higher than that observed following HLE monotherapy. These effects likely contribute to the enhanced efficacy of albendazole while mitigating excessive immunopathology.

## Discussion

The current pharmacological management of alveolar echinococcosis relies almost exclusively on benzimidazole carbamates, most commonly albendazole and mebendazole. Although these drugs remain the standard of care, their therapeutic efficacy is limited by poor water solubility, low intestinal absorption, and limited penetration into cysts protected by the laminated layer. Their antiparasitic activity results primarily from the inhibition of β-tubulin subunit polymerisation leading to disruption of energy metabolism and inhibition of parasite growth. However, because albendazole can also affect proliferating host cells [47], including immune cells, prolonged chemotherapy may further contribute to the impaired immune responsiveness associated with chronic infection. Clinical studies have demonstrated heterogeneous responses to chemotherapy among patients with AE [48–50], and according to WHO-PNM criteria, outcomes range from regressive disease to stable infection or continued progression despite long-term chemotherapy [50,51].

These limitations highlight the need for adjunct therapeutic strategies capable of enhancing antiparasitic efficacy while restoring protective host immunity. Current therapeutic approaches for many chronic diseases increasingly involve immunotherapy, either by enhancing endogenous immune responses or by supplementing natural immunoregulatory mediators. In line with this trend, in the present study, we investigated the immunomodulatory effects of a human low molecular weight leukocyte extract (HLE) administered either alone or as an adjuvant to albendazole during experimental sub-chronic alveolar echinococcosis in mice. In this model, a strong infection developed within four weeks post-inoculation in the peritoneal cavity, and two weeks of albendazole treatment produced a moderate, but significant reduction in parasite mass. HLE monotherapy also exerted measurable antiparasitic activity, consistent with our previous observations in experimental infection with the related cestode *Mesocestoides vogae* [13,14]. In the present work, HLE markedly potentiated the therapeutic efficacy of albendazole, with the combination treatment producing the greatest reduction in parasite burden. These findings are in agreement with the earlier study of Dvorožňáková et al. [24], who reported enhancement of albendazole efficacy in murine AE following administration of a comparable low molecular weight leukocyte extract prepared from porcine white blood cells (Imunor, AlphaMedica, CZ). Together, these observations support the concept that modulation of host immunity represents a promising strategy for improving the efficacy of conventional benzimidazole chemotherapy.

One of the principal findings of the present study is the marked reduction in circulating parasite-derived microRNAs following HLE treatment. MicroRNAs are highly conserved small non-coding RNAs that play critical roles in post-transcriptional regulation of almost all cellular signalling pathways in animals and plants by targeting specific mRNAs [52,53]. In *Echinococcus* spp., many miRNAs are widely expressed [54,55], and some are released into the host circulation either as free or encapsulated in extracellular vesicles (EVs), where they are thought to participate in host-parasite communication and modulate gene expression [33,56]. We therefore quantified four of the most abundant EV-associated parasite miRNAs (emu-miR-71a-5p, emu-let-7-5p, emu-miR-10a-5p and emu-miR-4989-3p) using RT-qPCR to assess whether therapies influenced their circulating levels. Examined miRNAs are believed to fulfil dual biological functions: they regulate parasite biology and development [55] and their secretion within EVs indicates an active role in shaping host–parasite interactions. For example, let-7 and miR-71 are highly abundant in *E. granulosus* and account for approximately 50% of total miRNA expression across developmental stages, suggesting essential roles in parasite development and survival within intermediate hosts through modulation of host immune cells [32,57]. In present study, albendazole therapy increased serum levels of miR-71a-5p, let-7-5p and miR-4989-3p, while decreasing miR-10a-5p relative to untreated mice. By contrast, HLE administered either alone or together with albendazole consistently reduced the circulating abundance of all four parasite-derived miRNAs. These distinct response patterns following ABZ monotherapy could potentially be attributed to drug-induced stress or damage to the metacestode tissue, leading to a transient release of vesicular contents into the circulation. This contrasts with the sustained suppression observed with HLE, suggesting the latter may act upstream in the miRNA biogenesis pathway.

While our data demonstrate a strong correlation between HLE treatment and reduced circulating parasite-derived miRNAs, the exact molecular mechanisms whether through direct inhibition of miRNA biogenesis within the parasite, disruption of EV packaging, or enhanced host clearance of these molecules-remain to be elucidated in future. In vitro experiments exposing *E. multilocularis* metacestodes to HLE, combined with analyses of extracellular vesicle production and miRNA processing pathways, will be necessary to distinguish between these possibilities.

Only a limited number of studies examined host target genes and signalling pathways targeted by helminth-derived miRNAs. Mortezaei et al. [58] demonstrated that exposure of *E. granulosus* protoscoleces to high concentrations of albendazole sulphoxide reduced let-7 expression in a dose-dependent manner. Cai et al. [59] reported that exosome containing *E. multilocularis* emu-miR-4989 were internalised by hepatocytes, where they inhibited the ubiquitin-conjugating enzyme UBE2N, a key regulator of TNF signalling and inflammation. Likewise, emu-miR-10a-5p packaged in *E. multilocularis* EVs reduced airway inflammation in allergic asthma by suppressing M2a macrophage accumulation [60], although its influence on M2 polarisation during AE may differ. Soichot et al. [61] reported that continuous exposure of macrophages to low levels of nematode *H. polygyrus* miR-71-5p dysregulated IFN-γ and mTOR signalling, with interferon regulatory factor 4 (IRF4) identified as a direct target. This miRNA also slightly increased IL-10 in alternatively activated macrophages. Together, these observations support the concept that parasite-derived miRNAs actively modulate host immune signalling pathways and thereby contribute to parasite persistence.

To determine whether the differential effects of albendazole and HLE on parasite-derived miRNAs were accompanied by changes in innate and adaptive immune responses, we examined cytokine production and cell populations in spleen, and the peritoneal cavity. Host immune responses to *E. multilocularis* involve a T cell-dependent process that includes sequential and overlapping Th1, Th2, Th17, and Treg involvement. The balance between these responses determines resistance, chronicity, or progression of infection [29]. In general, Th1-associated cytokines, particularly IFN-γ, IL-12 and IL-17 contribute to protective immunity and resistance, whereas Th2 cytokines and regulatory T cells (CD4 ⁺ CD25 ⁺ Foxp3⁺) promote tolerance through cytokines IL-4, IL-5, IL-10, IL-13 and TGF-β, thereby suppressing macrophage and dendritic cell maturation and reducing T cell proliferation. During the course of secondary *E. multilocularis* infection, host immunity gradually polarises towards an immunosuppressive T regulatory phenotype that favours long-term parasite persistence [28,29].

To date, little information exists regarding the influence of HLE on lymphocyte polarisation, function, and cytokine profiles during parasitic infection. In resent study, HLE administered alone or as adjuvant to ABZ markedly increased serum levels of the Th1 cytokines IFN-γ and IL-12, as well as the Th2 cytokine IL-4, while significantly reducing Treg- associated cytokines IL-10 and TGF-β. By comparison, albendazole monotherapy reduced only IL-10 and TGF-β without significantly enhancing Th1 cytokines. Polarisation towards a balanced Th1/Th2 response, accompanied with strong downregulation of T regulatory pathways was aligned with corresponding changes in lineage-defining transcription factors T-bet and GATA3 together with reduced Foxp3. In addition, HLE and combination therapy also downregulated expression of Atf3, a transcription factor associated with IL-10-producing regulatory B cells [41]. These findings suggest that HLE-induced modulation of T and B cell cytokine responses enhances albendazole’s antiparasitic efficacy. This interpretation is consistent with previous experimental and clinical observations. Depletion of Foxp3 regulatory T cells has been shown to improve control of *E.multilocularis* infection by promoting Th1/Th17 immunity [62]. Moreover, clinical studies in patients with related *E.granulosus* infection have shown that successful therapy was accompanied by significant decrease of IL-10, IL-4 levels and moderate IFN-γ elevation [63,64].

The local immune environment within the peritoneal cavity responded differently from the systemic compartment. At the local site of infection albendazole monotherapy increased mRNA expression of IFN-γ, TNF-α, IL-5, IL-4, IL-13, and NF-κB in peritoneal exudate cells while reducing TGF-β and IL-10 expression. Conversely, HLE and combination therapy substantially suppressed the expression of Th2 and Treg-associated cytokines while maintaining moderate IFN-γ expression. However, the mechanisms how individual therapies modulate NF-κB -related genes and the downstream cytokine alterations needs further proteonomic analysis. Elevated TGF-β levels in the infected liver tissue, has previously been associated with active AE and contributed to immune tolerance and fibrosis [65]. This pattern suggests that HLE does not simply stimulate immune responses but rather promotes their functional rebalancing by enhancing systemic protective immunity while limiting excessive inflammation at the site of infection.

We further assessed whether treatment-induced changes in parasite-derived miRNAs and cytokines levels were associated with alterations in proportions and functions of lymphoid and myeloid cell populations. Flow cytometric analysis revealed a profound expansion of CD11b⁺ myeloid cells in both the spleen and peritoneal cavity of infected mice, accompanied by a marked reduction in lymphoid populations and impaired T- and B-cell proliferative responses. Helminth infections typically induce strong inflammatory responses characterised by expansion of myeloid-derived suppressor cells (MDSCs) [66]. MDSCs are a heterogeneous population of immature CD11b⁺Gr-1 ⁺ cells comprising Ly6G⁺ polymorphonuclear and Ly6C⁺ monocytic subsets. However, Ly6C and Ly6G expression is not restricted to MDSCs [67]. Albendazole treatment partially reduced proportions of myeloid cells, whereas HLE administered alone or combined with albendazole, reduced myeloid cell frequencies by approximately 50%. This decline coincided with increased CD3^+^T cells, CD19^+^B cells and restored T and B cell proliferative responses detected ex vivo, indicating the reversal of helminth-induced immunosuppression. This interpretation is consistent with recent studies showing that T cell exhaustion mediated via TIGIT receptor [68] and expansion of tolerogenic regulatory T cells are major contributors to immune dysfunction during AE [69].

Further phenotypic characterisation of myeloid cells demonstrated comparable treatment-induced changes in both spleen and peritoneal cavity. Mature macrophages (CD11b⁺CD68 ⁺ MHCII^high^), likely of the M1 phenotype, remained relatively stable across experimental groups. In contrast, macrophages expressing low levels of MHC II, corresponding to the suppressive M2 phenotype, were highly abundant in infected mice but strongly reduced by HLE and combination therapy. Similar accumulation of M2 macrophages (MHC II ^low^) has been observed in hepatic lessions during late-stage murine infection [70]. Analysis of MDSC populations revealed elevated CD11b^high^Gr-1^high^ and CD11b^mid/high^ Gr-1 ^low/mid^ subsets in infected mice. Both subsets were significantly reduced by all therapies, particularly in mice treated with HLE and combination treatment, what is consistent with reduced MHC II^low^ macrophages. Populations of CD11b⁺Gr-1 ⁺ cells comprise both Ly6C⁺ monocytic and Ly6G⁺ granulocytic subsets. Similar suppressive MDSC expressing Ly6C and Ly6G with minimal MHC II expression have been described during related murine *E. granulosus* infection, where they exerted immunosuppressive functions on the Th2 lineage in a nitric oxide-dependent manner [71]. Immature CD11b⁺SiglecF^low^ eosinophils were similarly reduced following therapy, whereas mature CD11b⁺SiglecF^high^ eosinophils remained low in all groups, consistent with reduced eosinophilia typically associated with metacestode infection [29].

It is widely accepted that parasite excretory/secretory products drive MDSC expansion during helminth infections. We previously demonstrated that repeated intraperitoneal administration of *M. vogae* excretory products induced marked MDSC expansion in mice [72]. Likewise, antigen B secreted by *E. granulosus* has been shown to bind macrophages and modulate their function towards ineffective phenotypes [73]. The pronounced reduction in MDSCs observed following HLE treatment, together with the marked decline in circulating parasite-derived miRNAs, raises the possibility that HLE indirectly limits the parasite-derived signals responsible for sustaining suppressive myelopoiesis. The strong reduction of parasite-derived EV-associated miRNAs suggests that they can play role in maintaining suppressive myeloid and lymphoid responses. However, the present study does not distinguish whether these effects result from reduced secretion of parasite-derived immunomodulatory molecules, altered extracellular vesicle biology, or enhanced immune-mediated parasite damage. Future mechanistic studies will therefore be required to resolve these possibilities. However, albendazole effects likely result from direct damage to parasite tissue liberating larval antigens. Nevertheless, our findings are consistent with recent study of Zhang et al. [74] showing that targeting MDSCs promotes antiparasitic T cell immunity and enhances immune checkpoint PD-1 blockade efficacy during murine AE.

The reduction in suppressive myeloid populations was further supported by functional analyses of macrophage activity. Nitric oxide (NO) production by peritoneal exudate cells after ex vivo LPS stimulation was significantly decreased following HLE monotherapy and combination therapy.Elevated NO production is well-established feature of experimental AE [46] and has been implicated in the profound suppression of splenic lymphocyte proliferation through inducible nitric oxide synthase (iNOS)-dependent mechanisms [75]. The concomitant reduction in nitric oxide production and restoration of T- and B-cell proliferative responses observed in the present study therefore provide additional evidence that HLE alleviates parasite-induced immune suppression by limiting the accumulation and functional activity of suppressive myeloid cells.

### Conclusions

Albendazole therapy moderately but significantly reduced cyst mass, selectively altered circulating parasite-derived miRNA levels, moderately decreased IL-10 and TGF-β and myeloid derived suppressor cells in spleens and peritoneal cavities without effects on T and B cells proliferation suppression and NO production inhibition. HLE administered either as a monotherapy or in combination, profoundly suppressed serum levels of the major *Echinococcus*-derived miRNAs and these changes were accompanied by a marked restoration of host immune responsiveness, including a shift towards a balanced Th1/Th2 profile, suppression of regulatory cytokines, attenuation of immunosuppressive myeloid and MHC II ^low^ macrophage populations, and recovery of T and B lymphocyte proliferation. The combined therapy most effectively reduced parasite burden, correlating with the greatest normalisation of innate and adaptive immune parameters. Collectively, these results highlight that (1) HLE acts as a novel immunoadjuvant, with a mechanism involving downregulation of parasite-derived EV-miRNAs and suppression of the Treg/MDSC axis; (2) this combination strategy overcomes the immunosuppressive side effects of ABZ.

## Supporting information

S1 Table List of primers with their sequences and annealing temperatures used in RT-qPCR reactions. (DOCX)

S1 Raw Images Immunoblot analysis of transcription factors in spleens. (ZIP)
