# Supplementary material for: Human leukocyte extract enhances albendazole efficacy against Echinococcus multilocularis in mice by reducing parasitic microRNAs, myeloid and lymphoid immunosuppressive cells
Source: PLoS Negl Trop Dis. 2026 Aug 14;20(8):e0014591. doi: 10.1371/journal.pntd.0014591 (PMC13475980; doi:10.1371/journal.pntd.0014591)
Supplement: Supplementary file 1 [file pntd.0014591.s001.docx]

| Gene | Orientation | Sequence | Anneling temperature (°C) /time (sec) |
| --- | --- | --- | --- |
| GAPDH | forward | 5′-AGGTCGGTGTGAACGGATTTG-3‘ | 55.5 / 30 |
|  | reverse | 5′-TGTAGACCATGTAGTTGAGGTCA-3‘ |  |
| T-bet | forward | 5′-GCCAGGGAACCGCTTATATG-3′ | 55.5 / 30 |
|  | reverse | 5′-TGGAGAGACTGCAGGACGAT-3′ |  |
| GATA3 | forward | 5′-GAAGGCATCCAGACCCGAAAC-3′ | 56.0 / 40 |
|  | reverse | 5′-ACCCATGGCGGTGACCATGC-3′ |  |
| Foxp3 | forward | 5′-AATAGTTCCTTCCCAGAG-3′ | 56.2 / 40 |
|  | reverse | 5′-GATTTCATTGAGTGTCCT-3′ |  |
| CD9 | forward | 5´- CTC ATGATGCTGGTTGGTTTCC-3´ | 57.0 / 40 |
|  | reverse | 5´- CTCTAGACCATTTCTCGGCTC C-3´ |  |
| Atf3 | forward | 5- AGCCTGGAGCAAAATGATGCT T-3´ | 54.0 / 45 |
|  | reverse | 5- AGGTTAGCAAAATCCTCAAACAC-3´ |  |
| IFN-γ | forward | 5´-TCA AGT GGC ATA GAT GTG GAA GAA-3´ | 55.5 / 30 |
|  | reverse | 5´-TGG CTC TGC AGG ATT TTC ATG-3´ |  |
| TNF-α | forward | 5´-GGG ACA GTG ACC TGG ACT GT-3´ | 57.5 / 30 |
|  | reverse | 5´-GCA GAG GTT GAG TGA TGT AG-3´ |  |
| IL-5 | forward | 5´-TCACCGAGCTCTGTTGACAA-3´ | 55.0 / 30 |
|  | reverse | 5´-CCACACTTCTCTTTTTGGCG-3´ |  |
| IL-4 | forward | 5´-TCAACCCCCAGCTAGTTGTC-3´ | 55.0 / 30 |
|  | reverse | 5´-TTCAAGCATGGAGTTTTCCC-3´ |  |
| TGF-β | forward | 5´-TGACGTCACTGGAGTTGTACGG-3´ | 56.0 / 30 |
|  | reverse | 5´-GGTTCATGTCATGGATGGTGC-3´ |  |
| IL-10 | forward | 5- CGG GAA AGA CAA TAA CTG CAC CC-3´ | 57.0 / 40 |
|  | reverse | 5´- CGG TTA GCA GTA TGT TGT CCA GC-3´ |  |
| IL-13 | forward | 5-GGAGCTGAGCAACATCACACA-3´ | 56.5 / 30 |
|  | reverse | 5´-GGTCCTGTAGATGGCATTGCA-3´ |  |
| NFκB p65 | forward | 5´-GACCAACAATAACCCCTTTCAC-3´ | 57.0 / 30 |
|  | reverse | 5´-GTTTGAGATCTGCCCTGATGG-3´ |  |

Table 1: List of oligonucleotides and their sequences used in RT-PCR reactions
